# The *Healthy Native Youth Implementation Toolbox*: Using Implementation Mapping to adapt an online decision support system to promote culturally-relevant sexual health education for American Indian and Alaska Native youth

**DOI:** 10.3389/fpubh.2022.889924

**Published:** 2022-10-31

**Authors:** Christine M. Markham, Stephanie Craig Rushing, Jane Manthei, Michelle Singer, Cornelia Jessen, Gwenda Gorman, Melissa F. Peskin, Belinda F. Hernandez, Lea Sacca, Gabrielle S. Evans, Claudia Luna-Meza, Zoe Merritt, Ross Shegog

**Affiliations:** ^1^Center for Health Promotion and Prevention Research, University of Texas Health Science Center Houston, Houston, TX, United States; ^2^Northwest Portland Area Indian Health Board, Portland, OR, United States; ^3^Alaska Native Tribal Health Consortium, Anchorage, AK, United States; ^4^Inter Tribal Council of Arizona, Inc., Phoenix, AZ, United States; ^5^Center for Health Promotion and Prevention Research, University of Texas Health Science Center Houston, San Antonio, TX, United States; ^6^College of Medicine, Florida International University, Miami, FL, United States

**Keywords:** adolescent, sexual health promotion, American Indian and Alaska Native, interventions, dissemination and implementation research, Implementation Mapping

## Abstract

**Background:**

American Indian and Alaska Native (AI/AN) youth experience serious disparities in sexual and reproductive health, including the highest teen birth rate among racial/ethnic groups, and disproportionate rates of sexually transmitted infections (STI), including HIV. A growing number of evidence-based programs (EBPs) that integrate the strengths and cultural teachings of Native communities exist. Yet, multiple factors, including lack of trained personnel, limited resources, and geographic isolation, may hinder their adoption and implementation. Innovative implementation strategies that facilitate the adoption and implementation of sexual health EBPs in Native communities may help reduce these disparities.

**Methods:**

We applied Implementation Mapping, a systematic planning framework that utilizes theory, empirical evidence, and community input, to adapt a theory-based, online decision support system, iCHAMPSS (CHoosing And Maintaining Effective Programs for Sex Education in Schools), to support underlying dissemination and implementation processes unique to Native communities. We used an iterative design process, incorporating input from Native practitioners and academicians, to ensure that the adapted decision support system reflects cultural identification, community values, and experiences.

**Results:**

Grounded in diffusion of innovations, organizational stage theory, and social cognitive theory, the *Healthy Native Youth Implementation Toolbox* supports Native practitioners through five phases (Gather, Choose, Prepare, Implement, and Grow) to adopt, implement, and maintain a culturally-relevant, age-appropriate sexual health EBP. The *Toolbox* provides tools, ready-to-use templates, and guidance to plan, implement, and grow a culturally-relevant adolescent health program with their Tribe or community. Hosted within the Healthy Native Youth website (www.healthynativeyouth.org), the *Toolbox* comprises: (1) a curriculum portal with access to 15 culturally-relevant, age-appropriate evidence-based health promotion programs for AI/AN youth; (2) a “resource library” comprising 20+ support tools, templates, and links to external resources, and (3) “stories from the field” comprising testimonials from experienced Native educators, who have implemented sexual health programs.

**Conclusion:**

There is a continued need to design, test, and evaluate D&I strategies that are relevant to Native communities. The *Healthy Native Youth Implementation Toolbox* contributes to the dissemination and implementation of evidence-based, culturally-relevant sexual health education programs in diverse Native communities. Implementation Mapping provided a systematic approach to guide the adaptation process and integrate community voice with the ultimate goal of enhancing sexual health equity among AI/AN youth.

## Introduction

The federal government recognizes 574 distinct American Indian/Alaska Native (AI/AN) tribes that represent 2% of the United States (U.S.) population (1). Overall, the AI/AN population is young, with 30% under 18 years-old compared to 24% of the U.S. total population (2). As a result, the need for adolescent health promotion resources is particularly relevant in Native communities.

Despite recent declines in teen birth rates in the U.S., racial and ethnic disparities persist (3). AI/AN females ages 15–19 years have the highest teen birth rate among racial/ethnic groups (3) and the highest repeat teen birth rate (4). AI/AN youth are also disproportionately affected by sexually transmitted infections (STI), including HIV (5, 6). These health disparities may be ameliorated by the implementation of effective, culturally-relevant sexual health education programs (7). A growing number of evidence-based programs (EBPs) (8) that integrate the strengths and cultural teachings of Native communities have been developed or adapted for AI/AN youth (9–14). In 2016, our research team, in collaboration with AI/AN advisors, developed the Healthy Native Youth website (www.healthynativeyouth.org) to increase access to these culturally-relevant EBPs (15). The portal allows users to filter and compare curricula on multiple dimensions to determine best-fit and includes implementation materials free-of-charge. Yet, solely increasing access to culturally-relevant EBPs may be insufficient to increase their use (7). Multiple barriers exist and AI/AN health educators often lack the resources to navigate the adoption and implementation process. Adolescent sexual health is a sensitive topic, and many Native communities lack the community readiness and resources to broach the issue. Varying Tribal review and school board approval processes may create delays in program adoption and implementation (16). Pervasive poverty often results in personnel turnover or temporary closures for AI/AN youth-serving agencies, which may compromise implementation fidelity and program sustainability (7). Geographic challenges, including remote villages and reservations, may impact program implementation and access to resources (1, 7). Finally, as in other locations, AI/AN communities may face competing priorities, perceived lack of administrative or parental support, and lack of specialized training in sexual health, including limited knowledge of where to find culturally-relevant EBPs or limited self-efficacy to implement them (17, 18). Innovative strategies that facilitate the adoption and implementation of sexual health EBPs in Native communities are needed to reduce these health disparities.

iCHAMPSS (**CH**oosing **A**nd **M**aintaining Effective **P**rograms for **S**ex Education in **S**chools) is a theory-based online decision support system designed to address barriers to the dissemination and implementation (D&I) of sexual health EBPs in Texas schools (17, 19). Decision support systems are computer-based systems designed to facilitate a wide variety of decision tasks, including information gathering and analysis, alternative evaluation, and decision implementation (20). Grounded in D&I theories (21–23), iCHAMPSS comprises 60+ tools to provide step-by-step guidance to overcome D&I barriers for sexual education (www.ichampss.org). Demonstrated to impact critical determinants for adopting and implementing a sexual health EBP in Texas schools (24), iCHAMPSS serves as a promising implementation strategy to adapt for AI/AN communities.

To explore the potential of adapting iCHAMPSS, we conducted usability testing with AI/AN practitioners (*n* = 36) across the U.S. Overall, participants rated iCHAMPSS as acceptable, easy to use, credible, appealing, more helpful than current resources, and impactful of EBP adoption, implementation, and sustainability (25). However, using iCHAMPSS also significantly increased participants' perceived barriers to adopting an EBP. Some participants found the amount of information overwhelming and certain steps and tools, such as presenting a School Health Advisory Council (SHAC) recommendation letter to the School Board, were unfamiliar for Native communities. Sexual health education occurs in diverse settings in AI/AN communities, including schools, after-school programs, clinics, and community centers. Thus, the steps involved in the adoption and implementation of sexual health EBPs in Texas schools may not adequately reflect the steps involved in Native communities. Qualitative feedback from the usability testing provided tangible adaptation recommendations such as inclusion of culturally-relevant EBPs, provision of culturally appropriate assessment tools, integration of Tribal review and approval processes, and resources to adapt EBPs (25). Previous studies in AI/AN communities also suggest that embedding implementation within a consortium or learning community may enhance sustainability (26). Overall, findings indicated the potential for an adapted iCHAMPSS to address D&I barriers for sexual health EBPs in AI/AN communities.

In this “Methods” paper we describe how we applied Implementation Mapping to adapt iCHAMPSS to facilitate the adoption and implementation of sexual health EBPs in AI/AN communities. Implementation Mapping is a systematic approach for developing or adapting strategies to increase the adoption, implementation, and maintenance of evidence-based interventions, practices, or policies (27). It provides a step-by-step process, based in theory, empirical evidence, and community input, to identify the relevant determinants, mechanisms, and strategies for effecting change. The resulting *Healthy Native Youth Implementation Toolbox* (www.healthynativeyouth.org/implementation-toolbox/) is an online implementation strategy to increase the adoption and implementation of culturally-relevant, age-appropriate sexual health EBPs in Native communities with the ultimate goal of improving sexual health equity among AI/AN youth.

## Methods

### iCHAMPSS decision support system

**Figure 1:**
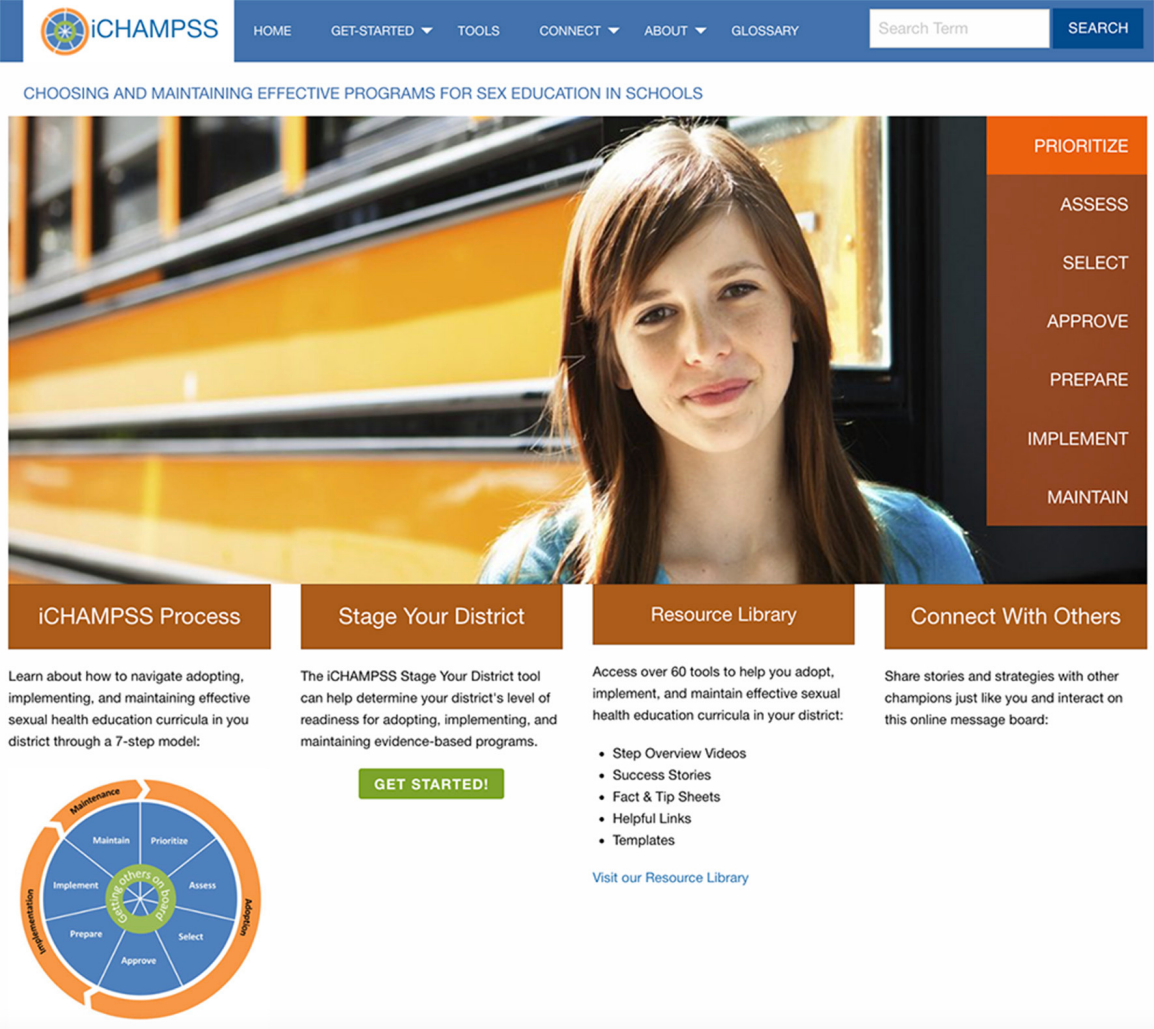
The i**CH**oosing **A**nd **M**aintaining Effective **P**rograms for **S**ex Education in **S**chools (iCHAMPSS) online decision support system.

iCHAMPSS is a web-based, interactive, self-paced decision support system that guides individuals through the process of adopting, implementing, and maintaining sexual health EBPs in Texas schools (Figure 1). iCHAMPSS comprises: (1) a “staging tool” to provide tailored guidance based on a community's level of readiness to implement a sexual health EBP, and (2) a “resource tools library” comprising 60+ support tools to enable successful completion of tasks within each implementation step. Tools include: step overviews, success stories (video testimonials from individuals who have adopted, implemented, or maintained a sexual health education EBP), facts and tips (e.g., a selection guide to identify EBPs), helpful links to online resources outside of iCHAMPSS, and templates that can be tailored to fit a school's or community's needs (19).

**Figure 2:**
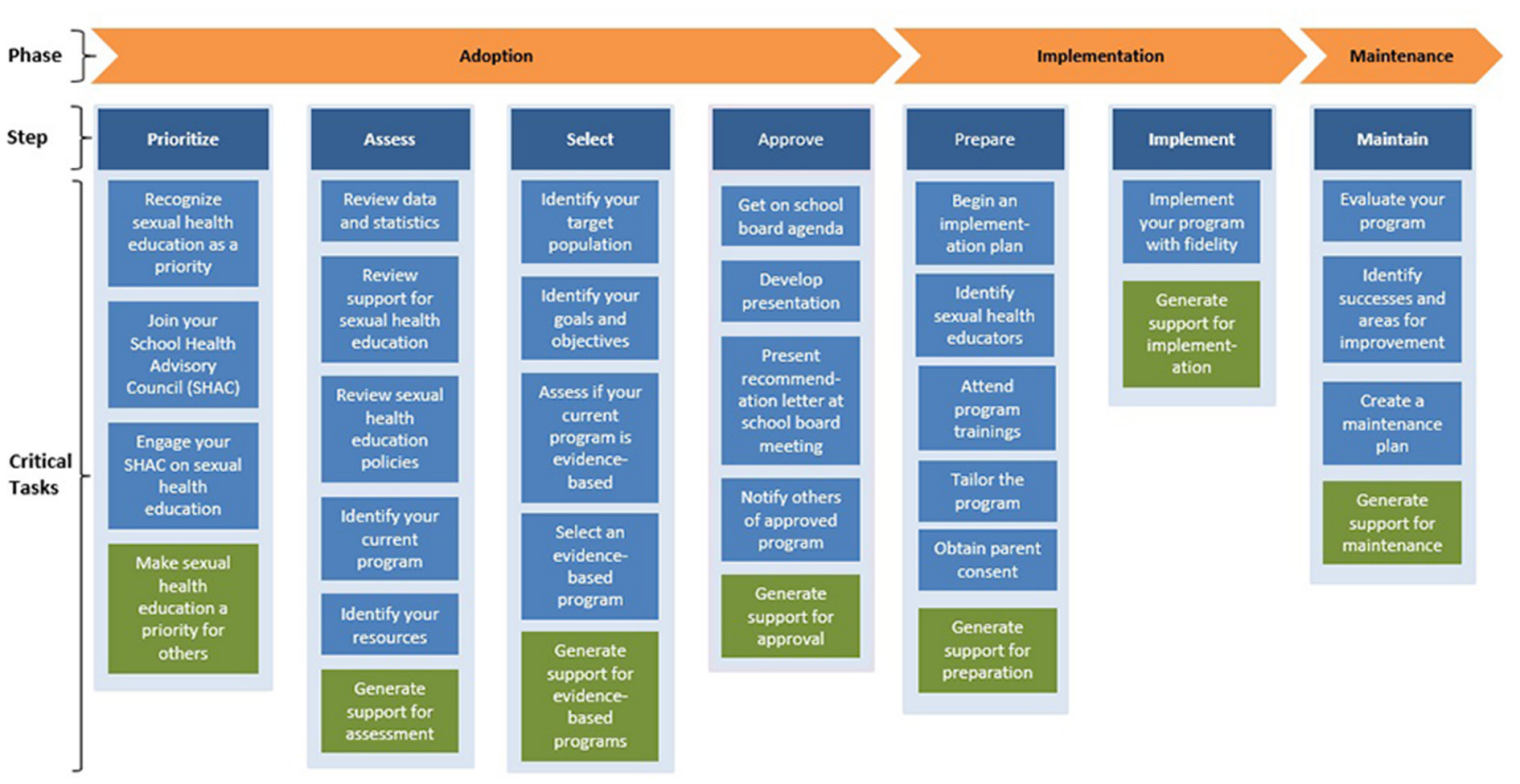
Phases, steps, and critical tasks in the CHAMPSS model (19).

**Figure 3:**
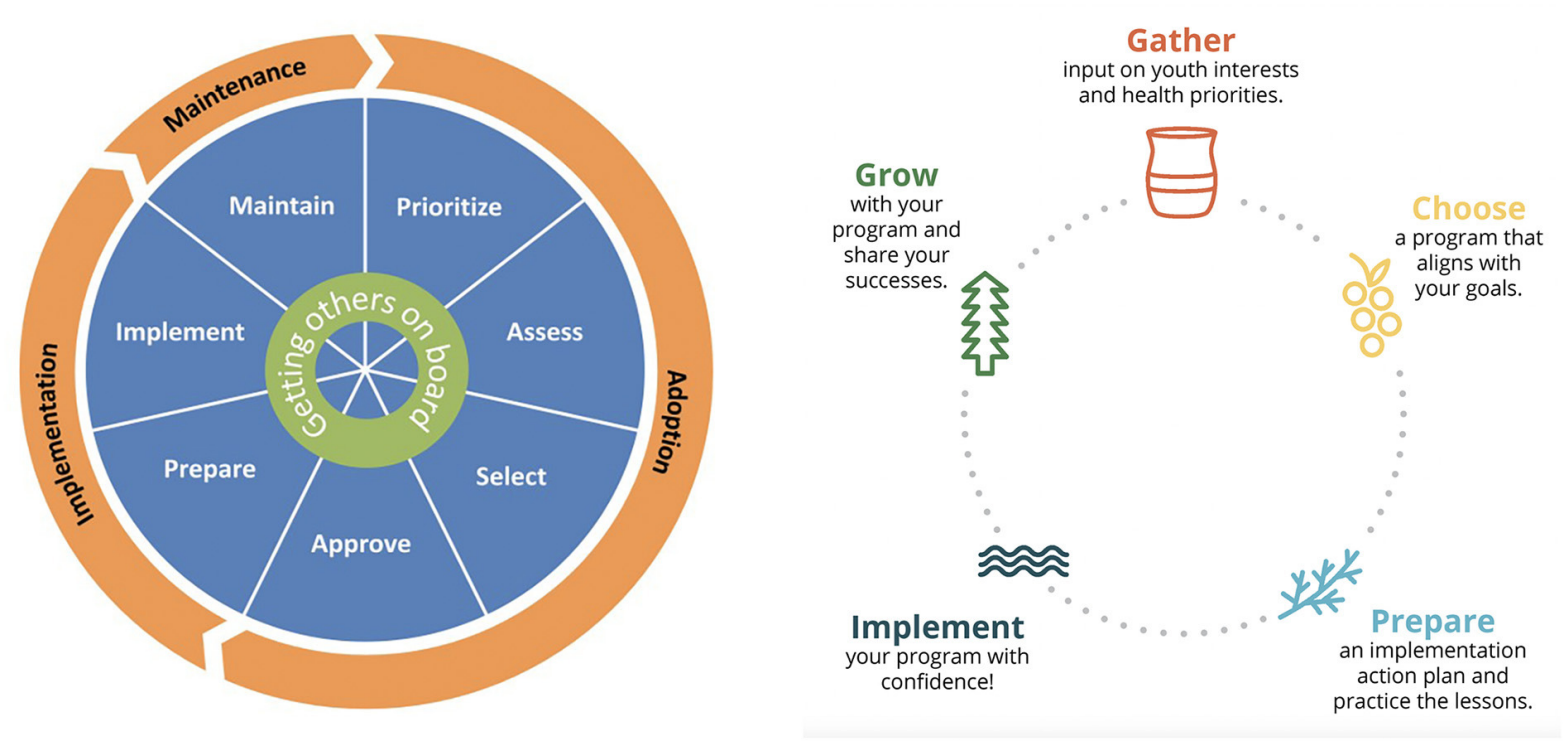
Original CHAMPSS and adapted *Healthy Native Youth Implementation Toolbox* models.

iCHAMPSS was developed using the original Intervention Mapping process (28). Guided by Diffusion of Innovation (21), Organizational Stage Theory (22), and Social Cognitive Theory (23), literature review findings on individual- and organizational-level factors that influence the adoption and implementation of sexual health EBPs in schools, and in-depth interviews with school district personnel, the research team developed adoption, implementation, and maintenance outcomes and performance objectives to delineate the specific actions needed to support sexual health EBPs in Texas schools. The resulting conceptual model, CHAMPSS (**CH**oosing **A**nd **M**aintaining Effective **P**rograms for **S**ex Education in **S**chools), provides the theoretical foundation for the web-based iCHAMPSS, and includes three phases: “adoption,” “implementation,” and “maintenance,” which are further divided into seven steps: (1) prioritize, (2) assess, (3) select, (4) approve, (5) prepare, (6) implement, and (7) maintain EBPs. A core element, “Generate support” (i.e., connecting with other supporters of EBPs and adolescent sexual health), extends across all seven steps. Each step comprises two to six sub-steps or critical tasks to move program planners through the process (Figure 2) (17). The model is circular (Figure 3), reflecting that planners may enter the model at any step, depending on their level of readiness. They may also complete one step but then realize they need to revisit a previous step.

iCHAMPSS incorporates theory-based methods and implementation strategies as step-specific tools to influence the determinants of adoption and implementation. For example, our success story video testimonials use modeling to influence planners' knowledge, skills, and self-efficacy to adopt, implement, or maintain a sexual health EBP. A detailed description of the development process is described elsewhere (17, 19).

The CHAMPSS model extends previous dissemination pragmatic models and frameworks (29–34) by providing greater focus on individual- and organizational-level determinants for the adoption, implementation, and maintenance of sexual health EBPs, and greater detail by operationalizing the steps needed to adopt, implement, and maintain sexual health EBPs in schools. The result is a pragmatic model with greater utility for practitioners, which is a recognized “model practice” by the National Association of County and City Health Officials (35).

### Participatory planning approach

Community-based Participatory Research Planning (CBPR) is an important component of Implementation Mapping. CBPR principles involve engaging with community partners to better understand the complex intervention context and to facilitate integration of real-world and academic knowledge to increase the potential effectiveness of interventions and implementation strategies (27, 36). Participatory planning is especially important in partnering with AI/AN communities to ensure the integration of Native-informed practice models and conceptual frameworks (37–39). The core planning group for the adaptation process comprised adolescent health educators and researchers at the Northwest Portland Area Indian Health Board (NPAIHB), the Alaska Native Tribal Health Consortium (ANTHC), the Inter Tribal Council of Arizona, Inc. (ITCA), and the University of Texas Health Science Center (UTHealth); hereafter, referred to as “we”. This group has collaborated for over a decade to adapt and develop online interventions and resources to promote adolescent sexual health in Native communities (11, 40, 41), including the Healthy Native Youth website (www.healthynativeyouth.org), which provides a “one-stop-shop” for Tribal youth advocates to access culturally-relevant curricula and resources (15). The Healthy Native Youth team also hosts monthly Community of Practice virtual gatherings to share resources with Native practitioners.

We used an iterative design process incorporating input from Native practitioners and academicians, to ensure that the adapted decision support system reflects cultural identification, community values, and experiences. During the planning phase (Implementation Mapping Tasks 1 and 2), we convened an Expert Advisory Group to provide high-level guidance on adaptation of the conceptual model and parameters for use for the adapted system. The group comprised researchers in Native adolescent health and representatives from Tribal Epidemiology Centers, the National Indian Health Board, the State of Alaska Adolescent Health Program, and other Native community-based organizations. During the design phase (Implementation Mapping Tasks 3 and 4), we conducted formative feedback sessions with our Healthy Native Youth AI/AN Adolescent Sexual Health Workgroup to obtain input on the adapted model, proposed tools, and website design mock-ups. The workgroup comprises Tribal health educators, advocates, teachers, counselors, academics, and representatives from additional national organizations including the United National Indian Tribal Youth, Inc. (UNITY), Big Brothers, Big Sisters, and Boys & Girls Club of America Native Services. As we began feasibility testing (Implementation Mapping Task 5), we solicited feedback on features and tools from each *Toolbox* phase during consecutive Healthy Native Youth Community of Practice sessions. Participants included Tribal health educators, teachers, parents, and prevention specialists. Overall, these groups met virtually online using Zoom software eight times between November 2020 and June 2022. We used interactive activities (e.g., Jamboard), chat feed discussions, and polling to obtain feedback on the adapted model, tools, and the website's features, tone, and feel.

### Implementation Mapping

Informed by the Intervention Mapping process and implementation science, Implementation Mapping provides step-by-step guidance for selecting, designing, or adapting implementation strategies to guide implementation efforts (27, 28). Implementation Mapping has been applied to improve the adoption, implementation, and sustainability of evidence-based programs, practices, and policies in real-world settings, including clinics, schools, and community-based service agencies (27, 42, 43). Implementation Mapping involves five specific tasks: (1) conduct a needs assessment and identify program adopters and implementers; (2) state adoption and implementation outcomes and performance objectives, identify determinants, and create matrices of change objectives; (3) choose theoretical methods and select or design implementation strategies; (4) produce implementation protocols and materials; and (5) evaluate implementation outcomes. These five tasks are iterative with the planning group circling back to previous tasks throughout to ensure all adopters and implementers, outcomes, determinants, and objectives are addressed (27). In this project, we applied Implementation Mapping (IM) to adapt iCHAMPSS to facilitate the adoption and implementation of culturally-relevant sexual health EBPs in AI/AN communities.

## Results

### IM Task 1. Conduct an implementation needs assessment

In IM Task 1, planners conduct a needs and assets assessment to identify barriers and facilitators of implementation. It is important to involve all agents including adopters, implementers, and those responsible for maintaining the evidence-based interventions to identify actions needed to implement the program and determinants (barriers and facilitators) of implementation (27).

To inform the adaptation process, we conducted a needs and asset assessment to identify barriers and facilitators for the adoption, implementation, and maintenance of sexual health EBPs in AI/AN communities. Given limited D&I research within Native communities, we conducted: (1) a broad scoping review to identify common barriers and effective implementation strategies to disseminate and implement health promotion EBPs in AI/AN, Native Hawaiian/Pacific Islander (NH/PI), and Canadian Indigenous communities, and (2) key informant interviews with experienced sexual health educators to identify factors specific to the D&I of sexual health EBPs in AI/AN communities.

#### Scoping review

Partnering with a research librarian, we identified research questions (What are the main barriers encountered in the D&I of EBPs in Indigenous communities? What implementation strategies have been used in Indigenous communities for EBP adoption, implementation and/or maintenance?), relevant electronic publication databases of PubMed, EMBASE, and Medline, formulated database search strategies, and developed a data abstraction form. To encompass a broad range of studies, EBPs were defined as any evidence-based or evidence-informed intervention or program disseminated or implemented in AI/AN, NH/PI, and/or Canadian Indigenous communities to improve health or behavioral outcomes for any age range. “Dissemination” and “Implementation” were defined in accordance with the 2016 National Institute of Health definitions (44). Barriers were classified into nine barrier categories within a broader socio-ecological framework (45). For comparability with D&I research in non-Indigenous communities, we categorized and coded implementation strategies according to the SISTER (School Implementation Strategies, Translating ERIC Resources) taxonomy of implementation strategies developed to facilitate the adoption, use, and maintenance of EBPs in school-based settings (46, 47). A detailed description of our scoping review methodology is described elsewhere (48).

Twenty-one studies met our inclusion criteria, representing community-based programs in diverse Tribal communities and settings. The programs encompassed a variety of health domains, including chronic disease and injury, substance misuse, wellness and illness prevention, and historical trauma, delivered among adults and/or children and youth. Key entities who were crucial to planning program implementation included decision makers in healthcare, school, community, organizations, academics, and government. Most cited barriers (*n* = 38) sorted into the category of “Social determinants of health,” which included barriers related to socioeconomic, geographic, and structural challenges, and the impact of historical oppression and trauma. Specific barriers related to program adoption included limited funding, competing demands, and lack of program integration with cultural values. These barriers created challenges in obtaining buy-in and support from key decision makers and community members. Barriers related to program implementation and maintenance included high attrition among program participants, high personnel turnover, limited evaluation skills among program implementers, and lack of technical assistance. These barriers have implications for ensuring implementation fidelity and sustaining community participation and support.

The most commonly reported SISTER implementation strategy (identified in 86% of studies) was: “Build partnerships (i.e., coalitions) to support implementation,” followed by “Capture and share local knowledge” (81%), “Tailor strategies” (71%), and “Conduct local consensus discussion” (52%). Four SISTER strategies, previously recognized as being *highly important* for D&I success in non-Indigenous settings were represented in the top 10 strategies (47). These were, “Conduct ongoing training,” “Monitor the progress of the implementation effort,” “Provide ongoing consultation/coaching,” and “Make training dynamic.” Four SISTER strategies previously described as *most feasible* for successful D&I in non-Indigenous settings were also represented in the top 10 (47). These were: “Capture and share local knowledge,” “Make training dynamic,” “Distribute educational materials,” and “Facilitation/Problem solving” (48).

#### Key informant interviews

NPAIHB, ANTHC, and ITCA team members invited five sexual health educators from their respective regions to share their experience adopting, implementing and maintaining sexual health education EBPs with AI/AN youth. The interviews were conducted *via* Zoom; they lasted about 45 min, and were audio-recorded for transcription. Participant characteristics were collected in a brief post-interview survey. Participants received a $20 e-gift certificate in appreciation of their time. We developed an interview guide based on the adoption, implementation, and maintenance steps outlined in the CHAMPSS model. Closing questions focused on recommendations to adapt iCHAMPSS for use in Native communities (see interview guide in Supplementary materials). For data analysis, we developed a codebook based on the interview guide to categorize each step in the adoption, implementation, and maintenance process as an analytic unit. We used ATLAS.ti to code the 15 key informant interviews according to the codebook. New codes were created based on emerging themes in each category and further broken down into subthemes.

Our key informants comprised nine women, three men, and one gender non-conforming individual. Two did not disclose their gender. The majority self-identified as AI/AN, with two also selecting Asian/Pacific Islander; four participants self-identified as non-Hispanic White. Five participants listed their primary role as a health educator; others included community representatives, clinical staff, a school administrator, youth mentor, and parent. Combined, participants had over 32 years' involvement in decision-making around or implementing sexual health education.

High rates of teen pregnancy and STIs were cited as key factors for prioritizing sexual health education in Native communities. Participants recommended engaging community partners, including community and Tribal leaders, elders, representatives from youth-serving agencies, parents, and youth throughout the planning process to build community support and reduce individual burden. Framing sexual health from a holistic health perspective and integrating culture as a protective factor helped to increase comfort and support for sexual health education. Compiling and sharing local data on adolescent health priorities and resources helped to generate support and guide program selection. Effective communication with key decision-makers, including Tribal Council and/or school board members, engaging youth voice, and preparing required paperwork, such as a memorandum of agreement, facilitated program approval.

Successful implementation of an approved program was influenced by the facilitator's community presence, visibility, and relationship with schools and community-based programs. Participants emphasized the need for pre-planning and effective communication with site leadership regarding program logistics (e.g., supplies, space, and co-facilitators) to avoid potential barriers. Integrating digital resources helped overcome geographic challenges. Effective teaching strategies included becoming comfortable with sexual health topics, being flexible, open-minded, culturally aware, and receptive to community and youth needs. Acknowledgment of diverse backgrounds and values within the classroom, encouraging youth voice, developing and enforcing classroom rules, and integrating self-care were identified as key factors for creating a supportive environment for facilitators and youth. Engaging youth as peer educators, providing incentives, and tailoring activities, such as inviting Tribal elders and clinicians as guest speakers, helped sustain youth involvement. Participants recommended engaging youth in reflecting on what worked well and what could be improved, and celebrating program successes with youth.

Successful maintenance of a sexual health program relied on ongoing, open communication with community members throughout the year. Sharing successes and lessons learned helped sustain interest and support. Seeking opportunities for community collaboration and input helped tailor programs to better reflect community-specific needs. Ongoing engagement with youth through cultural activities and events helped to maintain the excitement and “buy-in.” Given high personnel turnover, participants emphasized the need for ongoing training, technical assistance, and peer support to sustain and grow their program.

Recommendations for adapting iCHAMPSS for Native educators included greater representation of Native cultures and people through graphics, imagery, color schemes, and art. Participants appreciated the inclusion of videos to convey information, and recommended easy access to technical assistance or a program point of contact for implementation support. Overall, participants recommended simplifying the CHAMPSS model, and adapting the tools to reflect relevant processes in Native communities.

#### Prioritizing barriers and facilitators

With input from our Expert Advisory Group, the planning group synthesized findings from the needs and asset assessment to prioritize important and changeable barriers and facilitators for implementing culturally-relevant sexual health EBPs in AI/AN communities. Importance relates to how causally related a given barrier or facilitator is to implementation; changeability relates to the ease or difficulty of changing that factor (49). We chose to frame the prioritized list in the positive—that is, even when a barrier was identified, we stated it in terms of the change that needed to happen to improve implementation outcomes. We used these key recommendations to inform planning for IM Task 2 (Table 1).

**Table 1:** Implementation Mapping Task 1: Identified barriers and facilitators for adopting, implementing, and maintaining culturally-relevant, evidence-based sexual health education programs in AI/AN communities.

**Factors identified in the needs and asset assessment**	**Barrier**	**Facilitator**	**Source**		**Key recommendations**
			**Scoping review**	**Key informant interviews**	
**Adoption**					
Funding	✓		✓		• Engage community members, Tribal leaders, parents and youth in planning process • Obtain community input on adolescent health priorities and resources • Alleviate sensitivity by applying a holistic framework • Ensure cultural relevancy (in available EBPs and implementation support) • Communicate with key decision-makers
Competing demands	✓		✓	✓
Community partnerships		✓	✓	✓
Local knowledge		✓	✓	✓
Sensitivity of sexual health	✓			✓
Holistic health perspective		✓		✓
Cultural values		✓		✓	
Tribal council and school board approval processes	✓			✓
**Implementation**					
Socioeconomic, geographic, and structural challenges	✓		✓	✓	•Communicate with key decision-makers to overcome logistical challenges • Include digital channels to address geographic barriers • Provide appropriate training • Adapt program to fit local context and need • Engage youth in programming • Increase staff capacity to document implementation
Impact of historical oppression and trauma	✓		✓	✓
Level of comfort with sexual health topics	✓			✓
Participant attrition	✓		✓	✓
Responsiveness to youth and community needs		✓		✓
Tailored strategies		✓	✓	✓
Evaluation skills	✓		✓	
**Maintenance**					
Interest in program	✓		✓		•Communicate with community members • Collaborate with other youth programs • Provide ongoing training, technical assistance and peer support
Personnel turnover	✓		✓	✓
Community communication		✓		✓
Continued youth engagement		✓		✓
Training and technical assistance		✓	✓	✓

### IM Task 2. Identify adoption and implementation outcomes, performance objectives, determinants, and change objectives

In IM Task 2, implementation planners state adoption and implementation outcomes and performance objectives, identify determinants, and develop matrices of change objectives. Adoption and implementation outcomes are statements that describe the goal of program adoption, implementation, and maintenance. Performance objectives describe the specific steps, or sub-behaviors, that adopters and implementers must perform to meet that overall adoption or implementation goal. Performance objectives make clear “*who* has to do *what*” for the program to be adopted, implemented, and maintained. For example, for adopters, one question is: “*What do [adopters] have to do to make the decision to use [the program]?*” (27).

The planning group used findings from our needs and asset assessment and Expert Advisory Group feedback to adapt the adoption and implementation outcomes and performance objectives from the CHAMPSS model to better reflect the values and experiences of AI/AN communities. Table 2 lists the adoption and implementation outcomes and performance objectives for the *Healthy Native Youth Implementation Toolbox*.

**Table 2:** Implementation Mapping Task 2: Adapted adoption, implementation, and maintenance outcomes, actors, and performance objectives.

**Original iCHAMPSS phases**	**Adoption, implementation, and maintenance outcomes, and actors^a^**	**Performance objectives**
Adoption	GATHER community members to get guidance and feedback • AI/AN youth advocate(s) • Community partners	1. Connect with community members for guidance and feedback 2. Gather input on youth interests and health priorities 3. Identify your community's needs and resources 4. Select your program setting 5. Gather input from youth and program participants
	CHOOSE a culturally relevant health program and get approval if needed • AI/AN youth advocate(s) • Community partners	1. Identify decision-makers 2. Choose which criteria (e.g., participant age, setting, duration, and cost) are most critical 3. Select a program that aligns with your goals 4. Get approval, if needed 5. Seek input from youth and community
Implementation	PREPARE to implement a culturally relevant health program in your school or community setting • AI/AN youth advocate/s • Health educators • Peer educators • Community partners	1. Invite guest speakers 2. Attend Community of Practice sessions 3. Prepare an implementation action plan that includes self-care 4. Order supplies, teaching tools, and incentives 5. Practice going through the program and activities 6. Recruit caregivers, youth, and allies
	IMPLEMENT your program and celebrate the journey • AI/AN youth advocate(s) • Health educators • Peer educators • Community partners	1. Explore technical assistance and resource supports 2. Implement your program with confidence 3. Track your implementation journey 4. Assess student learning and experiences 5. Celebrate the youth
Maintenance	GROW and sustain your program • AI/AN youth advocate(s) • Health educators • Peer educators • Community partners	1. Collaborate with other youth programs 2. Grow with your program 3. Share successes and lessons learned 4. Keep the momentum going 5. Stay connected with youth beyond programming

a AI/AN youth advocates are typically representatives from school, afterschool, community-based, health, or clinic organizations; community partners include community and Tribal leaders, elders, representatives from other youth-serving agencies, parents, and youth.

Findings from Task 1 emphasized the importance of building partnerships, as well as capturing and sharing local knowledge, to support EBPs in AI/AN communities. Feedback from our Expert Advisory Group and key informant interviews reiterated the importance of collaborative processes, community involvement, and inclusion of youth voice throughout the planning process. Recommendations were to simplify the model, with a focus on community capacity-building and collective decision-making with the community and youth. Recognizing the diverse settings in which sexual health programs are implemented in AI/AN communities and the diverse profiles of Tribal health educators, we expanded key partners beyond the school system, and identified AI/AN youth advocate(s) (e.g., representatives from school, afterschool, community-based, health, or clinic organizations) and community members, including community and Tribal leaders, elders, representatives from youth-serving agencies, parents, and youth as key actors for program adoption, implementation, and maintenance. Additional actors for implementation include Tribal health educators and peer advocates for specific program delivery.

To simplify the tasks involved in program adoption, we combined two CHAMPSS' steps, “Prioritize” and “Assess,” into a single phase, titled “GATHER,” and two CHAMPSS' steps, “Select” and “Approve,” into a single phase, titled “CHOOSE.” GATHER recognizes the importance of community members coming together to share their learning, visionary wisdom, and perspectives. It recognizes Tribal communities as experts and engages with them as partners to gather input on adolescent health priorities and desired health skills. Taking a strengths-based, holistic approach, the model recognizes that adolescent sexual health represents one aspect of overall physical, mental, emotional, social, and spiritual health (50, 51). The GATHER phase performance objectives describe the specific steps that program adopters must take to identify youth interests and health priorities in their community.

“CHOOSE” recognizes the role of shared decision-making in selecting a health program that best aligns with these interests and health priorities. The CHOOSE phase performance objectives describe the steps that program adopters must take to select a culturally-relevant, age-appropriate, evidence-based health promotion program, and get approval from key decision-makers in a school or community setting, such as the school principal, clinic director, school board, health committee or Tribal council. Given varying tribal review and school board approval processes, these steps engage partners with decision-makers early in the planning process to better understand program constraints and requirements from their perspective.

For program implementation, we modified the CHAMPSS steps, “Prepare” and “Implement,” to help implementers plan and deliver a culturally-relevant program. The “PREPARE” phase performance objectives describe the steps needed to plan program implementation and gain support from key decision-makers. Inviting guest speakers, for example Tribal elders, recruiting peer educators, and integrating cultural activities, help to engage youth and community members, and increase program transparency. Integrating self-care planning for implementers and youth helps to reduce personnel burnout and create a supportive learning environment. “IMPLEMENT” focuses on program delivery with a shift from traditional fidelity and assessment to an emphasis on reflection, listening, and feedback. The IMPLEMENT phase performance objectives describe the steps needed to implement the program successfully and collect feedback to guide future program adjustments.

For program maintenance, we modified the CHAMPSS step, “Maintain,” to inform our “GROW” phase. “GROW” recognizes the importance of reflection and collaboration to nourish and sustain your program. The GROW phase performance objectives describe the steps that planners must take to grow and sustain their program by sharing successes with community members, and cultivating relationships across other youth programs and services to keep youth engaged.

The critical elements, “Get support” (i.e., connecting with other supporters of EBPs and adolescent health) and “Youth Voice” are integrated throughout the planning process in the first and final performance objectives of each phase. These elements underscore the importance and value placed in Native communities on building partnerships, capturing and sharing local knowledge, and ensuring inclusive participation throughout the adoption, implementation, and maintenance process.

**Figure 4:**
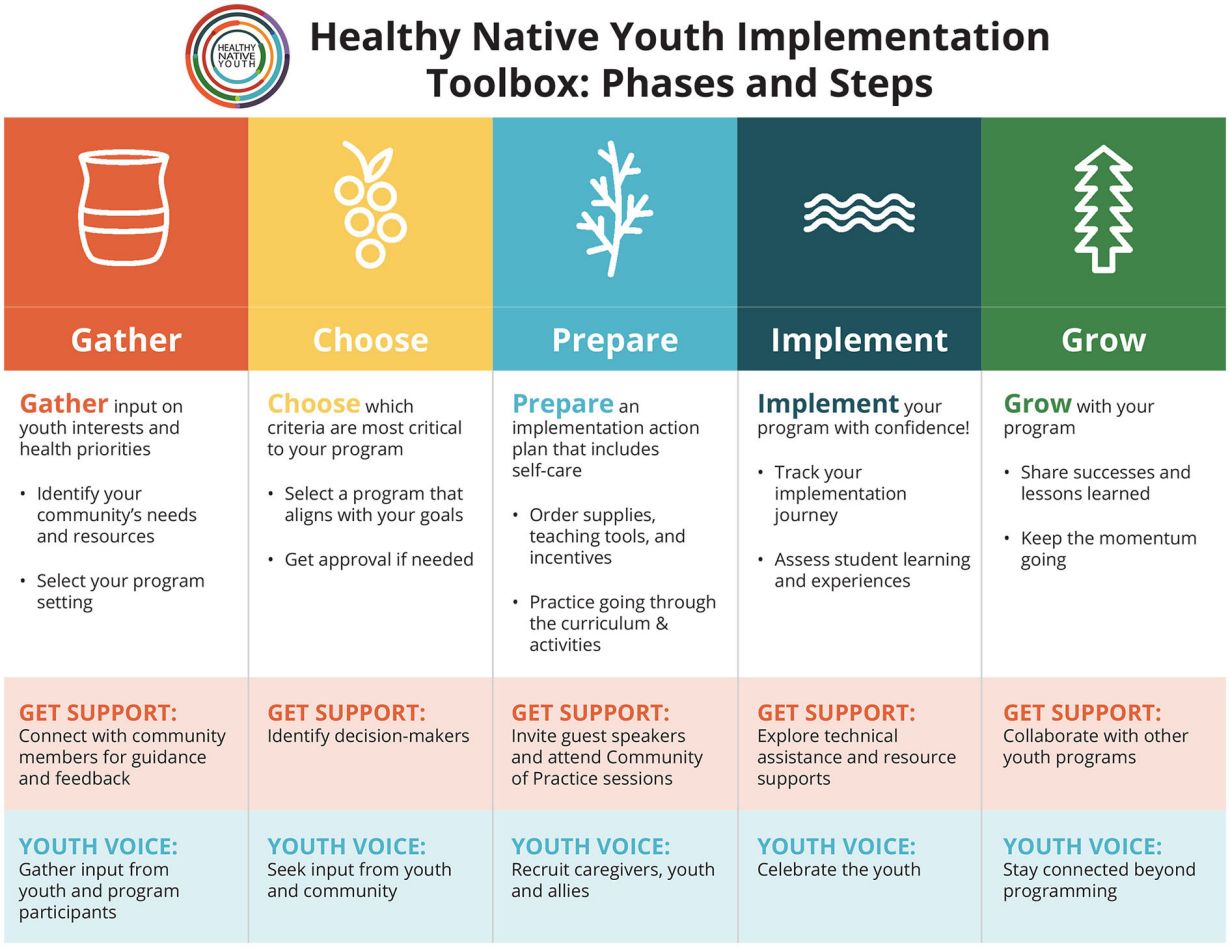
Phases and steps in the *Healthy Native Youth Implementation Toolbox* model.

As in the original CHAMPSS model, the *Toolbox* conceptual model is circular (Figure 3), indicating that partners may enter the model at any phase depending on their community's readiness or experience implementing sexual health EBPs, or they may enter the planning process at the beginning to adopt a new sexual health program. Figure 4 presents the “rolled-out” version of the model, illustrating the five phases (Gather, Choose, Prepare, Implement, and Grow) and phase-specific steps in the adapted *Healthy Native Youth Implementation Toolbox* model.

After we identified the performance objectives for each phase, we reviewed findings from Task 1 and the original CHAMPSS planning documents to identify important and changeable personal determinants for adopters and implementers. Determinants answer the question of “why?” For example, “*Why would an implementer deliver the program as planned*?” These may be constructs from health promotion theories, such as Social Cognitive Theory (23) or Theory of Planned Behavior/Reasoned Action Approach (52), or from implementation science frameworks. They are modifiable factors internal to the adopters and implementers that influence their adoption and implementation behavior (27). In developing the CHAMPSS model, we identified awareness/knowledge, attitudes, skills and self-efficacy, outcome expectations, and perceived norms as important and changeable determinants for sexual health EBP adopters and implementers (17, 19). The planning group agreed that these determinants were also relevant for adopters and implementers in AI/AN communities. Thus, we used these determinants to complete the final step in IM Task 2, develop matrices of change objectives.

Matrices cross performance objectives with personal determinants to produce change objectives. They answer the question: “What has to change in this determinant to bring about this performance objective?” Change objectives are the discrete changes required in each relevant determinant to influence achievement of the performance objective (27). Table 3 presents the matrix of change objectives for the Toolbox adoption phase, GATHER. The first performance objective is for the AI/AN youth advocate and community partners to “PO1: Connect with community members for guidance and feedback**”** and the corresponding change objective for the determinant, Awareness/Knowledge, is “List venues and organizations from which to engage youth and adult community members who understand and care about adolescent health priorities.” These matrices of change objectives formed the blueprints for adapting or developing new implementation methods and strategies in IM Task 3.

**Table 3:** Implementation Mapping Task 2: Example matrix of change objectives for adoption outcome, “GATHER community members to get guidance and feedback.”

	**Determinants**				
**Performance objectives (PO) AI/AN youth advocate and community partners^a^ will:**	**Awareness/knowledge (A/K)**	**Attitudes (A)**	**Skills and self-efficacy (SSE)**	**Outcome expectations (OE)**	**Perceived norms (PN)**
PO.1. Connect with community members for guidance and feedback	A/K.1.a. List venues and organizations from which to engage youth and adult community members who understand and care about adolescent health priorities	A.1.a. Recognize the value of integrating community voice, expertise, and resources throughout the planning process	SSE.1.a. Demonstrate ability to engage youth and adult community members in the planning process SSE1.b. Express confidence in building partnerships to help your program succeed	OE.1.a. Expect that obtaining guidance and feedback from youth and adult community members will help prioritize adolescent health issues in your community, and support implementation of your program	PN.1.a. Recognize that other youth advocates engage youth and adult community members in planning adolescent health programs
PO.2. Gather input on youth interests and health priorities	A/K.2.a. Describe different methods (surveys, social media poll, in-person interviews, Zoom breakout rooms, Poll feature) to assess adolescent health priorities and desired health skills	A.2.a. Feel positive about engaging youth and adult community members to identify youth interests and health priorities	SSE.2.a. Demonstrate ability to gather feedback from youth and adult community members SSE2.b. Express confidence to collectively identify youth interests and health priorities	OE.2.a. Expect that gathering input from different perspectives will help identify adolescent health priorities and desired health skills	PN.2.a. Recognize that other youth advocates and partners gather input to prioritize adolescent health topics
PO.3. Identify your community's needs and resources	A/K.3.a. Describe strategies to assess what youth, their families, and the broader community want to see in youth programming A/K.3.b. List available resources (staffing, program materials, teaching tools, funding) to implement an adolescent health program A/K.3.c. List constraints or challenges to be addressed A/K.3.d. List strategies to assess community readiness to inclusively address adolescent health, including needs of 2SLGBTQ youth	A.3.a. Feel positive about partnering with community members to identify needs and resources	SSE.3.a. Demonstrate ability to identify needs and resources for adolescent health programs SSE.3.b. Express confidence in assessing community readiness to inclusively address youth's health needs SSE.3.c. Express confidence in aligning adolescent health programs with community's cultural values and traditions	OE.3.a. State that identifying needs and resources for adolescent health will lead to adopting a program that is feasible, acceptable, and culturally relevant for youth in the community	PN.3.a. Recognize that youth advocates and partners in other communities assess needs and resources for adolescent health programs
PO.4. Select your program setting	A/K.4.a. List potential settings (e.g., school, afterschool, community, and clinic) to implement an adolescent health program A/K.4.b. List possible delivery modes (in-person, virtual, and hybrid) for adolescent health program A/K.4.c. Describe challenges or limitations (limited time, shared space, and few trained facilitators)		SSE.4.a. Express confidence in identifying potential settings and delivery modes for program implementation	OE.4.a. Describe how selection of potential settings and delivery modes by community partners will increase likelihood of successful program implementation	PN.4.a. Recognize that youth advocates and partners in other communities successfully implement adolescent health programs
PO.5. Gather input from youth and program participants	A/K.5.a. Describe how programs and services aimed at adolescents are likely to have a more significant impact if they are developed with the involvement of youth A/K.5.b. Describe ways to gather input from different youth audiences (rural, reservation, and urban) and age groups	A.5.a. Express that youth are experts on their own beliefs, values, and behaviors, as well as those of their peers	SSE.5.a. Demonstrate ability to gather youth input regarding program selection SSE.5.b. Express confidence in obtaining youth input in program selection	OE.5.a. State that obtaining youth input in the planning process will help ensure that selected program(s) are relevant to youth needs	PN.5.a. Recognize that youth advocates and partners in other communities value the inclusion of youth voice in decision-making

a AI/AN youth advocates are typically representatives from school, afterschool, community-based, health, or clinic organizations; community partners include community and Tribal leaders, elders, representatives from other youth-serving agencies, parents, and youth.

### IM Task 3. Select theoretical methods and design implementation strategies

In IM Task 3, planners choose theory- or evidence-based methods to in?uence the determinants identified in Task 2. They also select or design implementation strategies to operationalize those methods. Theory-based methods are techniques to influence determinants of implementation (27, 28). These methods can focus either at the individual level (the knowledge, attitudes, and skills of the implementer), or at the organizational level aimed at influencing organizational change directly (e.g., creating strong organizational leadership). Methods are important as they represent the underlying mechanism for change for an implementation strategy. Methods originate from behavioral, organizational, and community change theories, such as Social Cognitive Theory (23), the Elaboration Likelihood Model (53), Organizational Development Theory (54), and Models of Community Organization (55). These theories also specify “parameters” or situations under which a method is used appropriately. Implementation strategies refer to the ways in which program planners operationalize methods to influence determinants and change objectives for a specific adopter and task (small-scale strategies) or to the overall package of strategies influencing adoption, implementation, and maintenance (27).

In IM Task 3, we reviewed the theory-based methods and implementation strategies used in iCHAMPSS to guide decisions regarding the adaptation or development of culturally-relevant implementation strategies for the *Healthy Native Youth Implementation Toolbox*. During formative feedback sessions, our Healthy Native Youth AI/AN Adolescent Sexual Health Workgroup provided input on the acceptability and feasibility of specific implementation strategies to promote sexual health EBPs in Native communities.

In iCHAMPSS, we used multiple methods, including elaboration, persuasive communication, modeling, shifting perspective, goal-setting, and technical assistance to influence change objectives for the adoption, implementation, and maintenance of sexual health EBPs. The corresponding implementation strategies included step overviews, success stories, facts and tip sheets, ready-to-use templates, and helpful links (19). Reviewing these strategies, as well as existing culturally-relevant strategies developed by the planning group, such as the NPAIHB's Adolescent Health Tribal Action Plan (50), the *Healthy Native Youth: Virtual Adaptation Guide* (56), and strategies from the Native STAND Dissemination, Implementation and Evaluation project, we developed a list of possible methods and implementation strategies for the Toolbox. Table 4 provides examples of methods, parameters, and implementation strategies for steps in the GATHER phase. For example, in “PO.1. Get support: Connect with community members for guidance and feedback,” we used the methods of active learning (from the Elaboration Likelihood Model) (53) and enhancing network linkages (from Theories of Social Networks and Social Support) (57) to influence awareness/knowledge, skills, and self-efficacy related to connecting with community members. The associated implementation strategy was a customizable worksheet template to identify youth advocates and community partners.

**Table 4:** Partial Implementation Mapping Tasks 3 and 4: Steps, determinants, methods, parameters, implementation strategies, and example messages from the *Healthy Native Youth Implementation Toolbox* GATHER phase.

**GATHER steps^a^**	**Determinants and change objectives^b^**	**Methods^c^**	**Parameters^c^**	**Implementation strategies^c^**	**Example messages in the implementation strategy**
**Get Support:** Connect with community members for guidance and feedback	Awareness/knowledge A/K.1.a. Skills/self-efficacy SSE.1.a., 1.b. Attitudes, outcome expectations, and perceived norms A.1.a, OE.1.a PN.1.a.	Active learning Enhancing network linkages Persuasive communication Modeling	Requires time, information, and skills Requires available network Messages must be relevant, not too dissimilar from individual's beliefs Model must be relatable, describe specific steps or skills, receive reinforcement	Template: Customizable worksheet to identify youth advocates and community partners Phase overview: Supportive, friendly introduction to phase goal and steps Stories from the field: Video testimonial from a Native trusted advisor to inspire caring adults to support Native youth by selecting and implementing culturally-relevant programs	Teamwork makes the dream work! In the GATHER phase of the process, connect with community members to identify the health priorities and interests of youth in your program As you begin the planning process, it's a good idea to identify community partners that can support the delivery and implementation of your program
Gather input on youth interests and health priorities	Awareness/knowledge A/K.2.a. Skills/self-efficacy SSE.2.a., SSE.2.b. Attitudes, outcome expectations, and perceived norms A.2.a, OE.2.a PN.2.a.	Technical assistance Modeling	Must fit user's need, culture, and resources Model must be relatable, describe specific steps or skills, receive reinforcement	Helpful links: Links to example adolescent health action plans that incorporate adolescent health and wellness models Stories from the field: Video testimonial from an educator on the skills that Native youth learn from culturally-relevant programs	Engage diverse community partners to gather feedback from different perspectives to identify adolescent health priorities and desired health skills An educator shares her observation of Native youth learning accurate adolescent health information in their Native STAND class and paying it forward as peer educators
Identify your community's needs and resources	Awareness/knowledge A/K.3.a., A/K.3.b., A/K.3.c., A/K.3.d. Skills/self-efficacy SSE.3.a., SSE.3.b., SSE.3.c. Attitudes, outcome expectations, and perceived norms A.1.a OE.1.a PN.1.a.	Community assessment Community development	Requires assistance and possibilities for feedback Starting where the community is; may be grassroots or professional driven	Template: Customizable guide to conduct a community needs and resource assessment (who to engage, how to reach them, how and where to gather input, sample questions, how to share findings)	It is helpful to complete a community needs and resource assessment early in the planning process… This phase shouldn't be a major research effort! By gathering feedback or asking questions, you will be collecting valuable information and building partnerships that will help your program succeed.
Select your program setting	Awareness/knowledge A/K.4.a., A/K.4.b., A/K.4.c., Skills/self-efficacy SSE.4.a. Outcome expectations OE.4.a.	Active learning	Requires time, information, and skills	Template: Customizable worksheet to identify strengths and limitations of program settings and virtual platform options for adolescent health programs	Now, it's time to choose when and where to deliver the program… Think through each of your options: Will you implement the program in a school setting or a community setting? Will you deliver the program in-person, virtually, or in a hybrid manner?
**Youth Voice:** Gather youth input	Awareness/knowledge A/K.5.a., Skills/self-efficacy SSE.5.a., SSE.5.b. Attitudes, outcome expectations, and perceived norms A.5.a., OE.5.a., PN.5.a.	Active learning Participation	Requires time, information, and skills Requires willingness by the health promoter or convener to accept the participants as having a high level of influence	Activity guide: Interactive Bingo activity to make ensure programs reflect youth needs and concerns	Young people are experts on their own beliefs, values, and behaviors, as well as those of their peers. Consult with your Tribe's Youth Delegates, talk with your current students, or host a youth gathering and moderate the “Bingo Data Collection” activity to make sure your programs reflect their needs and concerns.

a Performance objectives from adoption outcome matrix for GATHER in Table 3.

b Determinants and change objectives from adoption outcome matrix for GATHER in Table 3.

c A theory-based method “is a general technique to influence determinants of implementation;” parameters are guidelines or conditions needed for a method to be effective; implementation strategies are strategies to influence specific determinants and change objectives of an adopter or implementer (27, 28).

After reviewing possible methods and implementation strategies for all five Toolbox phases with our Healthy Native Youth AI/AN Adolescent Sexual Health Workgroup, we identified a common set of implementation strategies or “tool types.” These included phase overviews, templates, examples, activity guides, helpful links to resources (including Healthy Native Youth Community of Practice recorded sessions), tips, and stories from the field (video testimonials from experienced AI/AN sexual health educators). Healthy Native Youth's Curriculum Portal and Request Technical Assistance feature were also identified as important implementation strategies. Table 5 provides a description of each “tool type,” including its related determinants, methods, purpose, and delivery mode.

**Table 5:** Implementation Mapping Tasks 3 and 4: *Healthy Native Youth Implementation Toolbox* tool types: Determinants, methods, delivery mode, purpose, and description.

**Tool types**	**Determinants**	**Methods**	**Delivery Mode**	**Purpose**	**Description and number of tools**
Phase overviews	Awareness/knowledge, attitudes, skills and self-efficacy, outcome expectations, and perceived norms	Persuasive communication	Text on screen	Introduction to the goal and steps of each phase	Supportive, friendly introductions to each phase's goal and steps (*n* = 5)
Templates	Awareness/knowledge, attitudes, skills and self-efficacy	Active learning	Customizable documents	Ready-to-use formatted examples of deliverables (e.g., community needs and resource assessment, letter of support, implementation action plan, attendance sheets)	Ready-made, easily modifiable documents that take the burden off the user (*n* = 17)
Examples	Awareness/knowledge, skills and self-efficacy, perceived norms	Modeling	Print materials	Sample models of deliverables (e.g., program budget, student surveys, certificate of completion, newspaper article)	Culturally-relevant, easy-to-replicate examples of print deliverables (*n* = 12)
Activity guides	Awareness/knowledge, attitudes, skills and self-efficacy	Active learning Participation	Print material	Guide for interactive feedback activity	Step-by-step guide for conducting interactive Bingo data collection activity (*n* = 1)
Helpful links	Awareness/knowledge, attitudes, skills and self-efficacy, outcome expectations, and perceived norms	Facilitation	Additional web resources	Credible / trustworthy outside resources for more information on particular topics	Easy-to-navigate links to resources including links to Community of Practice recorded session on HNY You Tube (*n* = 9)
Tips	Awareness/knowledge, skills and self-efficacy	Facilitation Persuasive communication	Text on screen	Encouraging advice for completing a particular phase	Tips and lessons from the field to assist with program selection, implementation, and growth (*n* = 7)
Stories from the field	Awareness/knowledge, attitudes, skills and self-efficacy, outcome expectations, and perceived norms	Modeling	Video	Stakeholder role models who validate the user's readiness and demonstrate how they successfully implemented a culturally-relevant adolescent health program	Stories from real practitioners who can relate their experience of changing attitudes and capabilities as they navigated barriers and achieved success (*n* = 7)
Healthy Native Youth curriculum portal	Awareness/knowledge, outcome expectations, skills and self-efficacy	Facilitation Technical assistance Active learning	Web-based resource	Preview and compare culturally-relevant, age-appropriate adolescent health curricula Access curricular materials and training	Culturally-relevant, evidence-based, age-appropriate adolescent health curricula on sexual health (*n* = 9), suicide prevention (*n* = 4), healthy coping (*n* = 1), and positive parenting (*n* =1) *Curriculum-specific program pages* provide information on training, lesson plans, supporting materials, cultural relevance, and evaluation findings (*n* = 15) *Curriculum comparison chart* allows user to compare curricula by criteria (e.g., age, setting, duration, cost, and evidence of effectiveness; *n* = 1)
Healthy Native Youth Community of Practice and recorded sessions	Awareness/knowledge, attitudes, skills and self-efficacy, outcome expectations, and perceived norms	Modeling Facilitation Technical assistance	Video	Online learning community to share experiences with AI/AN youth advocates. Users may review previously recorded Community of Practice sessions and download supporting documents	Community of Practice online sessions provide resources and opportunities to engage with Native professionals in AI/AN adolescent health (*n* = 42)
Healthy Native Youth request technical assistance form	Awareness/knowledge, attitudes, skills, and self-efficacy	Facilitation Technical assistance	In-person	Allows user to submit an online request for technical assistance or training to select or implementing a culturally-relevant program or address other youth topics	Online form to submit technical assistance request

### IM Task 4. Produce implementation protocols and materials

In IM Task 4, planners produce implementation protocols, activities and/or materials. Similar to Step 4 in Intervention Mapping, this requires planners to create design documents, draft content, pretest and refine content, and produce final materials. Design documents are shared between planners and production teams, and they are created for each document or other materials that are a part of the implementation strategy (27).

In Task 4, the planning group developed design documents and drafted content to guide production of the *Healthy Native Youth Implementation Toolbox* and its supporting tools. The design documents provided detailed instructions for program designers to produce the Toolbox, including specific content, messages, and tools for each Toolbox phase. We shared proposed tools and website design mock-ups with our Healthy Native Youth AI/AN Adolescent Sexual Health Workgroup to obtain feedback prior to final production.

#### Website development

We partnered with the original Healthy Native Youth website design team to develop and integrate the *Toolbox* into the existing website. Utilizing an user-centered design process, the website designers created “use cases” to determine different user experiences interacting with the Toolbox, and wire frames to guide feedback with the planning group during website development. To increase accessibility, the *Toolbox* is designed to be viewed on desktop, laptop, tablet, and mobile devices.

**Figure 5:**
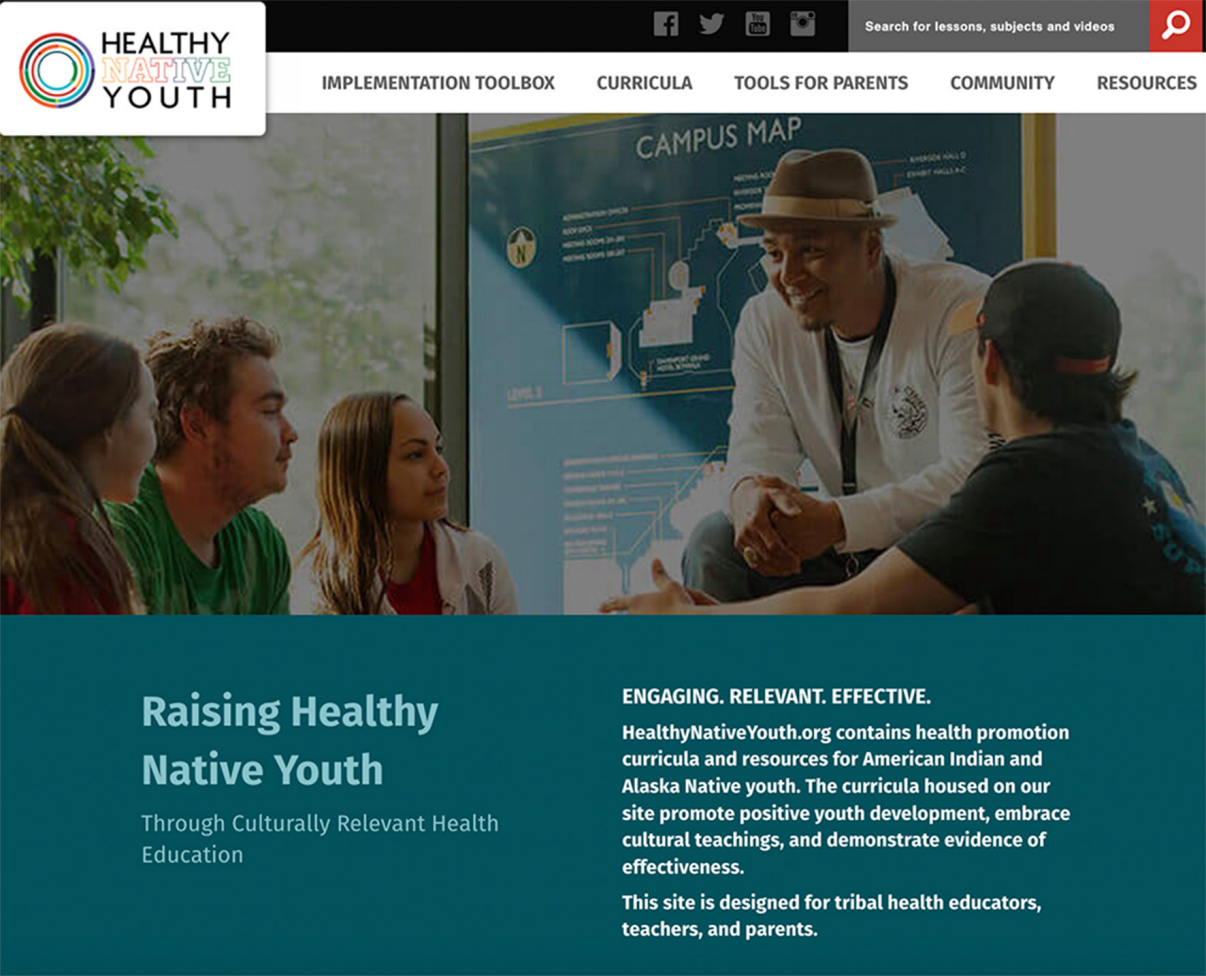
Screen capture of the Healthy Native Youth website home page.

**Figure 6:**
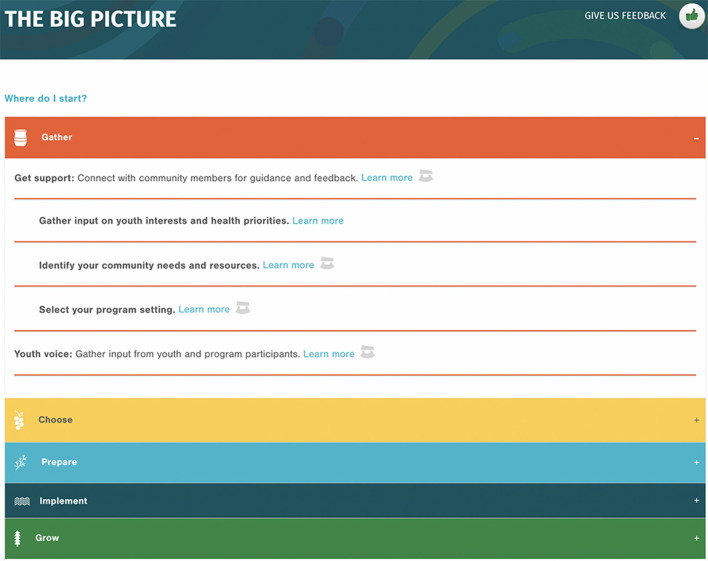
Screen capture of the *Healthy Native Youth Implementation Toolbox*: The Big Picture feature.

The *Implementation Toolbox* is accessed *via* the Healthy Native Youth website (www.healthynativeyouth.org; Figure 5). The home page includes links to an Introduction, which orients users to the purpose of the Toolbox, and two features, “Where Do I Start?” and “The Big Picture,” which are tailored to the user's experience or need. The “Where do I start?” feature is tailored for users who have already started the process of implementing youth programs and may have specific goals. The user can select from a list of nine activities, each relating to one of the five phases, such as, “I want to engage youth in the planning process,” “I want to do a community needs assessment,” or “I want to select a health curriculum,” and be directed to the relevant phase and tools. “The Big Picture” feature provides a concertina-style overview of the five phases and their respective steps and tools, so that users may select their own entry point into the *Toolbox*. The “ulu” icon (an all-purpose knife traditionally used by Inuit, Iñupiat, Yupik, and Aleut women) indicates links to relevant tools. “The Big Picture” feature was designed for easy viewing on mobile devices (Figure 6).

**Figure 7:**
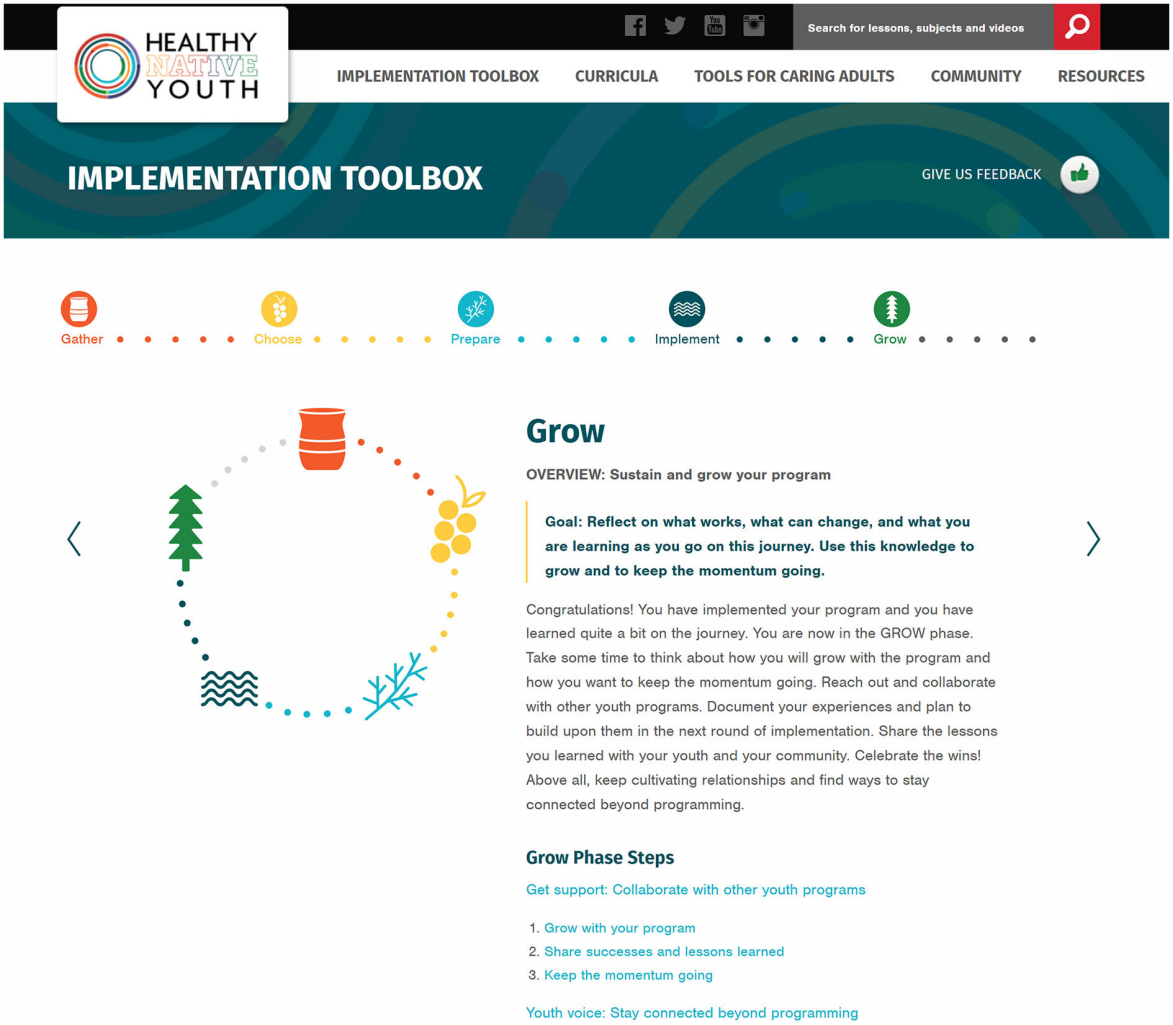
Screen capture of the *Healthy Native Youth Implementation Toolbox*: GROW phase overview.

Within the *Toolbox*, each phase has its own Phase Overview page that orients the user to the goal and steps for that phase, including steps for Get Support and Youth Voice. Each overview page leads to step-specific pages with links to relevant tools (templates, examples, activity guide, tips, helpful links, or success stories) to successfully complete the phase. A radio button panel across the top of each page indicates the user's overall progress through the phases and steps (Figure 7).

#### Tools development

**Figure 8:**
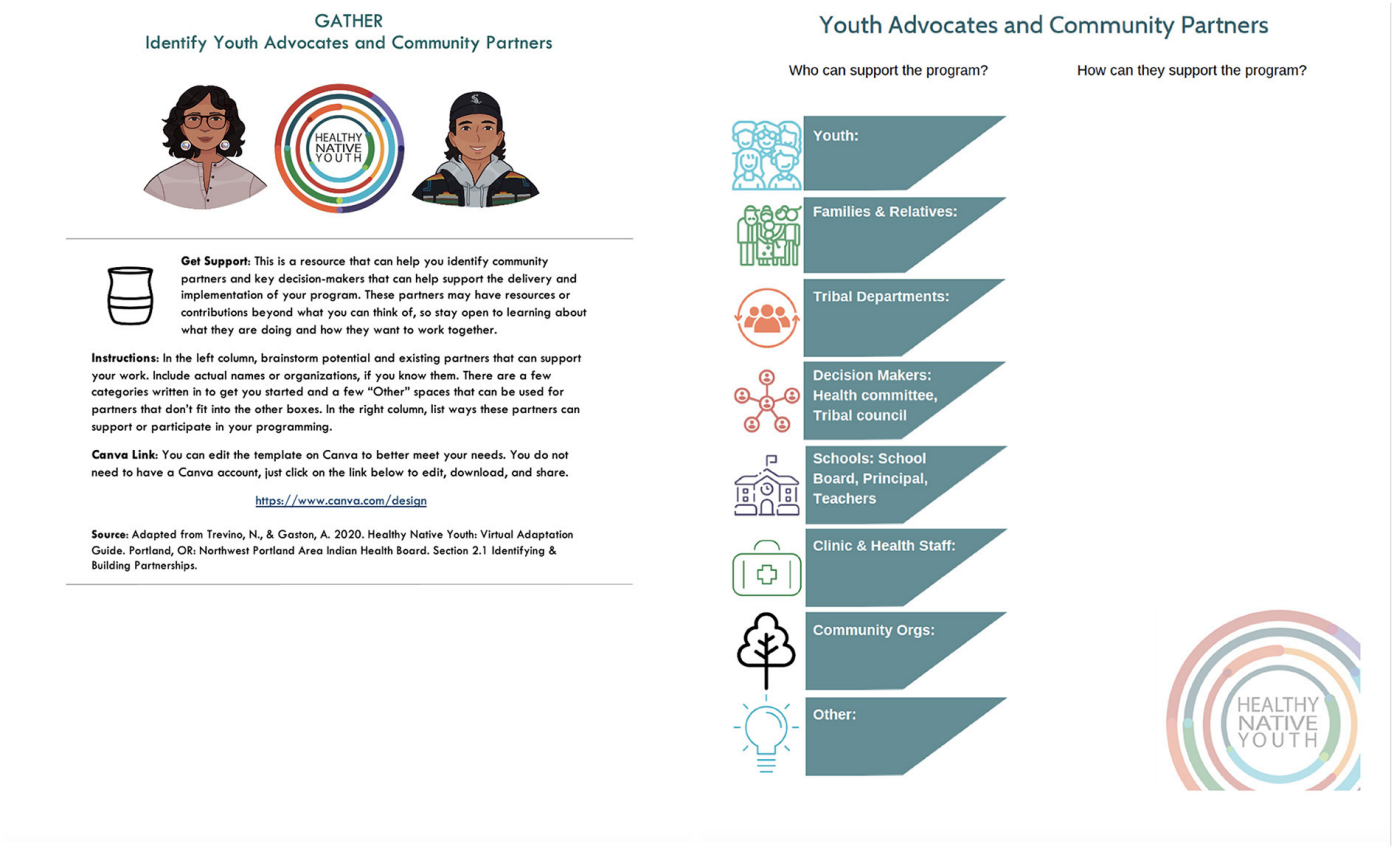
Template for GATHER phase: Youth advocates and community partners map.

The planning group developed design documents for each tool that specified its purpose, delivery mode, content, and messages. The tone of the messages is user-friendly, strength-based, and supportive. Table 3 (sixth column) provides example of specific messages for tools in the GATHER phase. The NPAIHB graphic design team developed Indigi-icons, reflective of Native values, to represent each phase in the planning process (Figure 4). Each tool includes the Indigi-icon for its respective phase and simple instructions on how to use the tool. Figure 8 presents the customizable template from the GATHER phase to create a youth advocates and community partners map.

**Figure 9:**
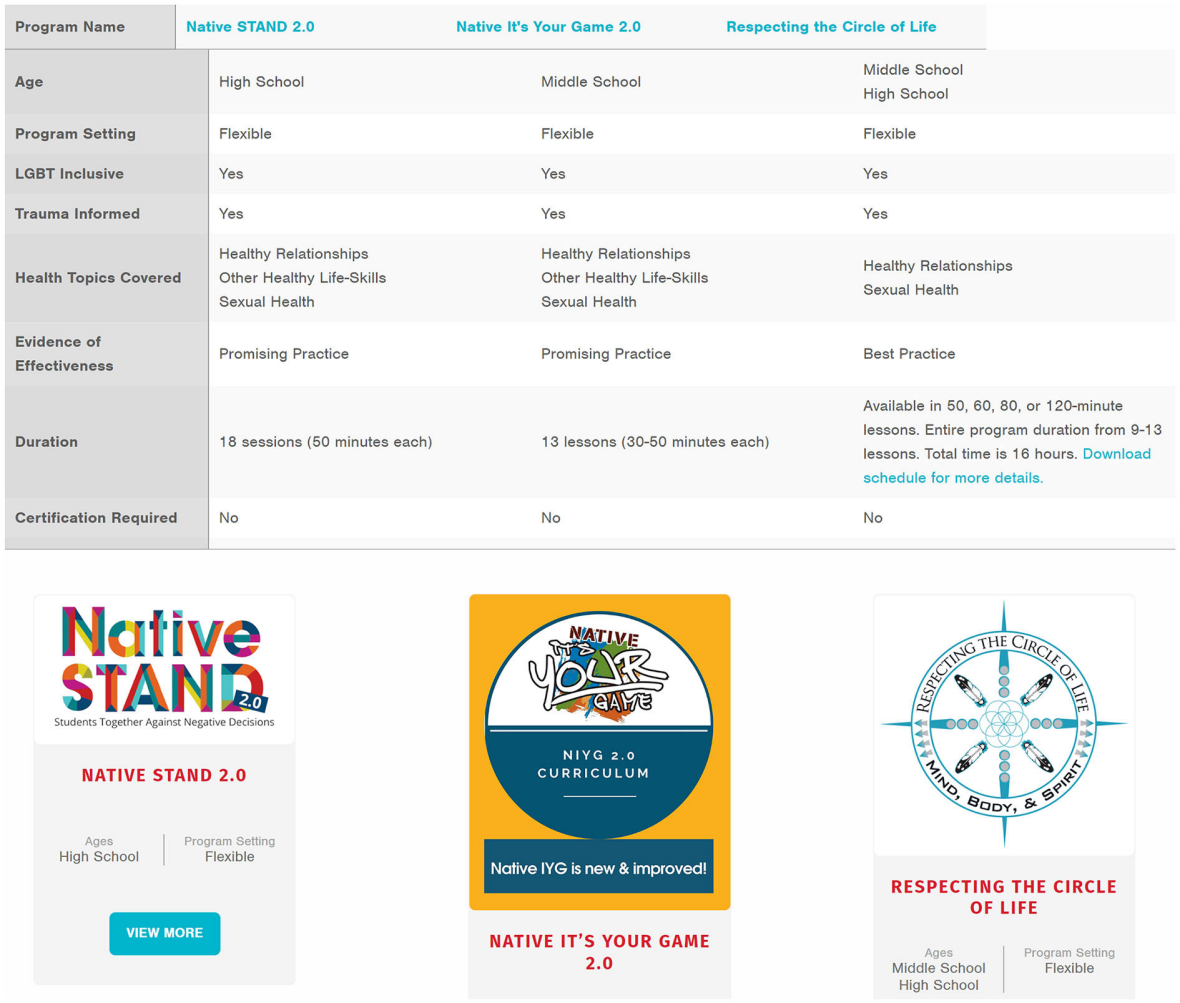
Healthy Native Youth curriculum portal resources, including curriculum comparison chart and example curricula.

Healthy Native Youth's *Curriculum Portal* is an important tool for the CHOOSE phase as it provides free access to culturally-relevant, age-appropriate evidence-based curricula designed or adapted for AI/AN youth. The portal currently includes nine curricula related to sexual health, four related to suicide prevention, and two related to healthy coping and positive. *Curriculum-specific program pages* provide information on training, lesson plans, supporting materials, cultural relevance, and evaluation findings. The *Curriculum Comparison Chart* allows users to compare curricula by criteria (e.g., age, setting, duration, cost, and evidence of effectiveness) to select a curriculum that best aligns with their community's goals. Evidence of effectiveness follows the Center for Disease Control and Prevention's (CDC) classification of evidence-based practices: emerging practice, promising practice, leading practice, best practice, and/or tribal best practice (8) (Figure 9).

Finally, the Healthy Native Youth Community of Practice and Request Technical Assistance features provide peer and technical support from the Healthy Native Youth Collective Partnership to help AI/AN youth advocates adopt and implement culturally-relevant health programs. The Healthy Native Youth SMS text messaging series provides additional resources for directly for youth and trusted adults. See Supplementary material for a comprehensive list of current *Toolbox* tools by phase. Table 6 provides a side-by-side summary of adaptations by Implementation Mapping task from the original iCHAMPSS decision support system to the adapted *Healthy Native Youth Implementation Toolbox*.

**Table 6:** Summary of Adaptations from iCHAMPSS to the *Healthy Native Youth Implementation Toolbox* by Implementation Mapping Task.

	**Original iCHAMPSS**	**Adapted Healthy Native Youth (HNY) Implementation Toolbox**
**IM Task 1. Conduct an implementation needs assessment**		
Priority population	• Texas school districts	• American Indian and Alaska Native (AI/AN) communities
Innovation being disseminated	• US DHHS recognized evidence-based sexual health education programs	• Culturally-relevant, evidence-based sexual health education programs
Stakeholder feedback groups	• School-based community stakeholder group (district level School Health Advisory Council [SHAC] members, district curriculum coordinators, school nurses, and parents)	• Expert advisory group of Native adolescent health researchers and practitioners • HNY AI/AN adolescent sexual health workgroup • HNY community of practice participants
Adopters	• School district level personnel (Board of Trustees and SHAC members), school principals	• AI/AN youth advocate(s) (e.g., representatives from school, afterschool, community-based, health, or clinic organizations) and community partners (community and Tribal leaders, elders, representatives from youth-serving agencies, parents, and youth)
Implementers	• District curriculum coordinator, school principals, school curriculum coordinator, and teachers	• AI/AN youth advocate(s), health educators, peer educators, and community partners
Maintainers	• District and school curriculum coordinators, principals, and teachers	• AI/AN youth advocate(s), health educators, peer educators, and community partners
**IM Task 2. Identify adoption and implementation outcomes and performance objectives**		
Conceptual model based on implementation outcomes and performance objectives	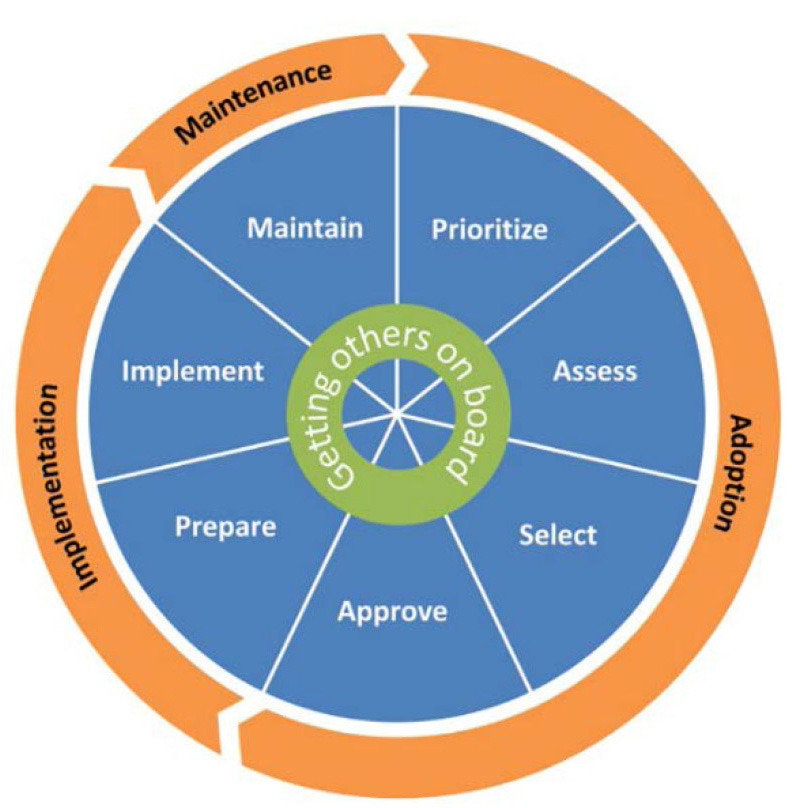	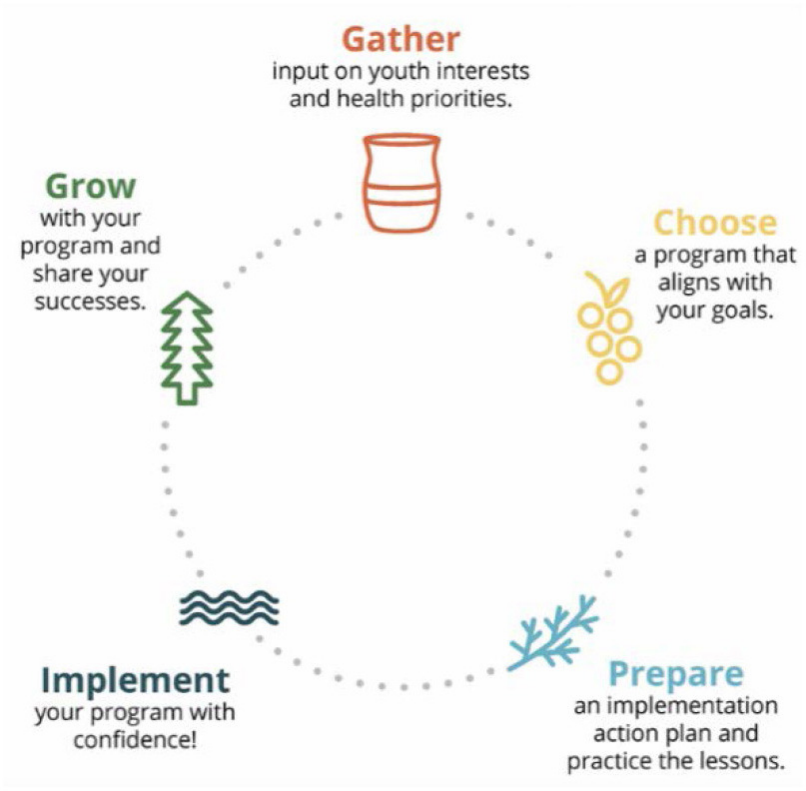
**IM Task 3. Select theoretical methods and design implementation strategies**		
Theoretical methods Implementation strategies (examples)	• Persuasive communication: Step overview videos • Modeling: Success story testimonial videos • Active learning: Templates • Technical assistance: Facts and tips • Technical assistance: Helpful links • Enhancing network linkages: Online message board	• Persuasive communication: Phase overview pages • Modeling: Stories from the field testimonial videos • Active learning: Templates and activity guides • Technical assistance: Helpful links • Technical assistance: Request technical assistance • Enhancing network linkages: HNY Community of Practice online sessions
**IM Task 4. Produce implementation protocols and materials**		
Website url	• www.ichampss.org	• www.healthynativeyouth.org
Delivery vehicle	• Desktop, laptop	• Desktop, laptop, tablet, and mobile devices
Point(s) of entry	• Get Started feature; Stage Your District tool	• The Big Picture and Where Do I Start? Features
Curriculum selection tools	• EBP Selection guide (pdf) lists US DHSS reviewed evidence-based sexual health education curricula (*n* = 26) by curriculum characteristics (e.g., age, gender, race/ethnicity, and outcomes of evaluation study, cost, training requirements)	• Curriculum portal provides access to culturally-relevant, evidence-based sexual health, substance use, suicide prevention, healthy coping, and positive parenting curricula (*n* = 15) • Curriculum Comparison Chart allows users to compare curricula by criteria (e.g., age, setting, duration, cost, and evidence of effectiveness)
Testimonial videos	• Success Stories from experienced Texas school district personnel	• Stories from the Field from experienced AI/AN sexual health educators
Tools library	• 60+ tools	• 20+ tools
Images	• School district and school settings, diverse youth and adults	• AI/AN communities, youth, adults, and elders • Indigi-icons
Communication and networking	• Online message board	• HNY Community of Practice online sessions
Technical assistance	• Contact us feature	• Request technical assistance feature • Recorded HNY Community of Practice sessions
**IM Task 5. Evaluate implementation outcomes**		
Preliminary evaluation	• Usability and pilot study with Texas school personnel	• Feasibility study with AI/AN youth advocates

We launched the *Healthy Native Youth Implementation Toolbox* in December, 2021. We have sequentially shared the GATHER, CHOOSE, PREPARE and IMPLEMENT, and GROW phase tools with AI/AN youth advocates at four, online Community of Practice sessions. The feedback from practitioners has been positive, with comments including: “very user friendly,” “helpful, easy to understand,” “concretely helpful tools,” “visually great,” “ease of access.” We also received feedback that, “Downloadable tools and templates in the Big Picture were hard to find.” We are also actively disseminating the *Toolbox via* Healthy Native Youth's e-newsletter, Twitter, and Facebook page and Indiancountryecho.org. Based on feedback, we are compiling a list of features for the *Healthy Native Youth Implementation Toolbox version 2.0*, which will include a searchable “tools library” to help users locate the tools they need.

### IM Task 5. Evaluate implementation outcomes

In IM Task 5, planners develop an evaluation plan that describes expected implementation outcomes for adoption, implementation, and/or maintenance (27). To inform evaluation planning, we are conducting a feasibility study to obtain feedback from AI/AN youth advocates on their experience using the *Toolbox*, and to assess its preliminary impact on individual and community-level determinants for implementing culturally-relevant sexual health EBPs in AI/AN communities. Using a convenience sample, pre/post-test design, we have recruited 29 individuals from AI/AN youth-serving organizations across the U.S. to trial the *Toolbox* for a 6-month period. We will use pre- and post-test survey data to assess changes in stage of community readiness to adopt/implement/maintain a sexual health EBP; individual knowledge and attitudes toward culturally-relevant sexual health EBPs; perceived support of an EBP by various groups (e.g., parents and Tribal leaders); self-efficacy to complete each *Toolbox* step, and network connections to advocate for culturally-relevant sexual health EBPs. Post-test survey items adapted from previous usability instruments will assess acceptability, ease of use, utility, credibility, motivational appeal, and perceived helpfulness (24, 25, 58, 59). Additional items request recommendations for future enhancements. Findings will inform the development of *Toolbox Version 2.0* and provide preliminary data for a future multisite effectiveness-implementation trial.

## Discussion

Limited tools exist to help AI/AN communities adopt, implement, and maintain culturally-relevant, age-appropriate, evidence-based adolescent sexual health education programs. We used the systematic planning approach, Implementation Mapping, to adapt an existing online decision support system, iCHAMPSS, to better support sexual health education D&I processes in Native communities. The resulting conceptual model that underlies the *Healthy Native Youth Implementation Toolbox* is reflective of the values and experiences of AI/AN communities. More importantly, the *Toolbox* provides guidance and decision support to Tribal health advocates on each phase of the process, sharing adaptable ready-to-use templates, relatable examples, and stories from the field. Many health educators tasked with selecting and implementing a culturally-relevant, age-appropriate sexual health program do not have formal training in public health or research methods. Developing approachable language and visuals, offered in phased bite-size pieces, is critical to meet the needs of diverse program champions, who in turn must navigate diverse delivery settings. Many of the tools and templates now featured in the *Toolbox* had already been used in the field by Tribal health educators, but were not logically sequenced or offered with accompanying tips or examples. Consolidating these tools and resources into a comprehensive *Toolbox* was a critical next-step to support AI/AN health advocates and community partners to navigate the planning process.

Using Implementation Mapping to guide the adaptation process had multiple advantages and helped address several challenges previously identified in the implementation science literature. Prior research has highlighted the need for methods that improve the selection and tailoring of implementation strategies for a given setting (60), and that articulate the causal pathways through which implementation strategies are effective (61). Implementation Mapping provided a systematic approach to select, adapt, and create implementation strategies that are tailored to the cultural values and realistic experiences of Native communities. In Tasks 1 and 2, we identified barriers and facilitators unique to the D&I process of sexual health EBPs in Native communities, and developed culturally-relevant behavioral outcomes and performance objectives to guide the adaptation process. In Tasks 3 and 4, we selected theory-based methods that would influence the personal determinants of Native adopters and implementers, and designed culturally-relevant tools and messages to facilitate the D&I process. The explicit linkage of determinants to methods to tools articulates the proposed mechanism of change that underlies the *Toolbox*.

Prior research has also highlighted the critical role of community engagement to accelerate and improve the implementation of EBPs. Community-engaged D&I research can help improve health inequities through incorporating unique perspectives from communities, that have been historically left out of the research process (62, 63). Collaborative planning is a fundamental principal of Implementation Mapping (27). Our adaption process involved a multi-disciplinary research team together with input from diverse partners ranging from national experts to educators on the ground to capture the unique experience of implementing sexual heath EBPs in Native communities. We are continuing to collect feedback from users to guide further development of the *Toolbox* to ensure higher reach, satisfaction, and sustained implementation outcomes. Continued training and technical assistance will be also critical to successfully support uptake and use.

Developing culturally-relevant implementation strategies requires collaboration with AI/AN practitioners and academicians, as well as responsiveness to Native-informed practice models and conceptual frameworks (37, 38). Interventions must also align with organizational capacity and community readiness to be sustainably implemented (39). Our adaptation process was informed by cultural sensitivity adaptation frameworks and principles (37, 64–68), and included changes to surface and deep structures (65). Surface structure adaptations involved matching materials and messages to observable characteristics of AI/AN communities (e.g., images, people, and locations), while deep structure involved incorporating cultural, social, environmental, and psychological processes unique to the dissemination and implementation of sexual health EBPs in Native communities. We used an iterative design process, incorporating input from diverse Native partners, to ensure that the final product reflects cultural identification, community values, and needs.

Although using Implementation Mapping had multiple advantages, it was not without its challenges. These included the time required to identify relevant outcomes and performance objectives that reflected the values and processes involved in adopting, implementing, and maintaining sexual health educations programs in Native communities. This process took over a year to complete, with iterative feedback from our advisory groups and community members. It then took 6 months to translate these objectives into supportive, accessible messaging and tools that would resonate with our intended audience. Lessons learned along the way included the critical role that NPAIHB, ANTHC, and ITCA's collective experience partnering with AI/AN communities played in grounding the adaptation process from a holistic, strengths-based perspective, and the importance of collaborating with experienced AI/AN creatives for website development and graphic design to ensure that *Toolbox* features, language, and imagery were relevant and engaging for Native practitioners.

Alongside these lessons learned, several limitations should be noted. First, the *Toolbox* represents an adaption of an existing online decision support system rather than the development of a new program using ethnographic and grounded theory approaches. Thus, it does not meet the ideal of a culturally-based, culturally congruent, and culturally grounded practice emerging from AI/AN world views (37). Second, for our scoping review, although the similarity with findings from previous studies indicates some validity across cultural settings, our coding, or limited D&I research in these settings, may have failed to identify implementation strategies that are unique to Native communities. Third, the limited practitioner sample for our key informant interviews and feedback during the adaptation process means that the generalizability of the conceptual model and implementation strategies are unknown. Finally, the feasibility and efficacy of the *Toolbox* are yet to be established. Findings from our feasibility study will provide feedback to further refine the *Toolbox*, and future studies should focus on a rigorous evaluation to assess its impact on the adoption, implementation, and maintenance of sexual health EBPs in Native communities.

## Conclusion

There is a continued need to design, test, and evaluate D&I strategies that are relevant to Native communities. The *Healthy Native Youth Implementation Toolbox* contributes to the dissemination and implementation of evidence-based, culturally-relevant sexual health education programs in diverse Native communities. The *Toolbox* moves beyond simply providing access to EBPs to help Native communities successfully navigate the adoption and implementation process. Implementation Mapping provided a systematic approach to guide the adaptation process and integrate community voice with the ultimate goal of improving sexual health equity among AI/AN youth.

## Data availability statement

The original contributions presented in the study are included in the article/Supplementary material. Further inquiries can be directed to the corresponding author.

## Ethics statement

The studies involving human participants were reviewed and approved by the Institutional Review Board at the University of Texas Health Science Center at Houston, Committee for the Protection of Human Subjects (CPHS), and the Alaska Area Institutional Review Board. Appropriate tribal approval was obtained in Alaska through the Alaska Native Tribal Health Consortium. Written informed consent for participation was not required for this study in accordance with the national legislation and the institutional requirements.

## Author contributions

All authors made substantial contributions to the conception, design of the work, acquisition, analysis, interpretation of the data, and drafted the work or critically revised it for important intellectual content, provided final approval of the manuscript, and agreed to be accountable to all aspects of the work.

## Funding

This work was funded by the Indian Health Service HIV Program and the Secretary's Minority AIDS Initiative Fund and by the National Institute on Minority Health and Health Disparities NIH/NIMHD 1R21MD013960-01A1. Native iCHAMPS: An Innovative Online Decision Support System for Increasing Implementation of Effective Sexual Health Education in Tribal Communities. PIs: CM, RS, and MP.

## Conflict of interest

The authors declare that the research was conducted in the absence of any commercial or financial relationship that could be construed as a potential conflict of interest.

## Publisher's note

All claims expressed in this article are solely those of the authors and do not necessarily represent those of their affiliated organizations, or those of the publisher, the editors and the reviewers. Any product that may be evaluated in this article, or claim that may be made by its manufacturer, is not guaranteed or endorsed by the publisher.

## References

[1] Office of Minority Health. Profile: American Indian/Alaska Native. (2022). Available online at: https://minorityhealth.hhs.gov/omh/browse.aspx?lvl=3&lvlid=62 (accessed February 27, 2022).

[2] U. S. Census Bureau. Facts for Features: American Indian and Alaska Native Heritage Month: November 2021. (2022). Available online at: https://www.census.gov/newsroom/facts-for-features/2021/aian-month.html (accessed February 27, 2022).

[3] Martin JA Hamilton BE Osterman MJK. Births in the United States, 2017. NCHS Data Brief. (2018) 318:1–8. 30156535

[4] Gavin LE Warner LS O'Neil ME Duong LM Marshall C Hastings PA . Vital signs: repeat births among teens — United States, 2007–2010. Morb Mortal Wkly Rep. (2013) 62:249–55. 23552226 PMC4605012

[5] Centers for Disease Control and Prevention. HIV in the United States by Race/Ethnicity: HIV Incidence. Available online at: https://www.cdc.gov/hiv/group/racialethnic/other-races/incidence.html (accessed August 22, 2022).

[6] Indian Health Service and Centers for Disease Control and Prevention. Indian Health Surveillance Report - Sexually Transmitted Diseases 2015. Rockville, MD: U.S. Department of Health and Human Services (2018).

[7] Kaufman CE Schwinn TM Black K Keane EM Big Crow CK. The promise of technology to advance rigorous evaluation of adolescent pregnancy prevention programs in American Indian and Alaska Native tribal communities. Am J Public Health. (2016) 106:S1:S18–20. 10.2105/AJPH.2016.303335 27689483 PMC5049466

[8] Spencer LM Schooley MW Anderson LA Kochtitzky CS DeGroff AS Devlin HM . Seeking best practices: a conceptual framework for planning and improving evidence-based practices. Prev Chronic Dis. (2013) 10:130186. 10.5888/pcd10.130186 24331280 PMC3864707

[9] Craig Rushing S Gaston A Kaufman C Markham C Jessen C Gorman G . Using technology to promote health and wellbeing among American Indian and Alaska Native teens and young adults. In: Dyson LE, Grant S, Hendricks M, editors, Indigenous People and Mobile Technologies. Routledge, Taylor & Francis Group. (2016). p. 163–78.

[10] Tingey L Chambers R Patel H Littlepage S Lee S Lee A . Prevention of sexually transmitted diseases and pregnancy prevention among Native American youths: a randomized controlled trial, 2016-2018. Am J Public Health. (2021) 111:1874–84. 10.2105/AJPH.2021.306447 34529503 PMC8561210

[11] Shegog R Craig Rushing S Jessen C Lane T Gorman G Torres J . Native It's Your Game: improving psychosocial protective factors for HIV/STI and teen pregnancy prevention among youth in American Indian/Alaska Native communities. J Appl Res Child. (2017) 8:3.

[12] Hafner SP Craig Rushing S. Sexual Health, STI and HIV risk, and risk perceptions among American Indian and Alaska Native emerging adults. Prev Sci. (2019) 20:331–41. 10.1007/s11121-018-0920-7 30006906

[13] Skye M McCoy T Kelley A Singer M Rushing SC Donald C . Effectiveness of Native STAND: a five-year study of a culturally relevant sexual health intervention. Adolescents. (2021) 1:321–34. 10.3390/adolescents1030024

[14] Kaufman CE Whitesell NR Keane EM Desserich JA Giago C Sam A . Effectiveness of circle of life, an HIV-preventive intervention for American Indian Middle School Youths: a group randomized trial in a Northern Plains Tribe. Am J Public Health. (2014) 104:e106–12. 10.2105/AJPH.2013.301822 24754555 PMC4062020

[15] Craig Rushing S Stephens D Shegog R Torres J Gorman G Jessen C . Healthy Native Youth: Improving access to effective, culturally-relevant sexual health curricula. Front Public Health. (2018) 6:225. 10.3389/fpubh.2018.00225 30175091 PMC6107849

[16] Jernigan VBB Jacob T Styne D. The adaptation and implementation of a community-based participatory research curriculum to build tribal research capacity. Am J Public Health. (2015) 105:S424–32. 10.2105/AJPH.2015.302674 25905848 PMC4455516

[17] Hernandez BF Peskin MF Shegog R Markham CM Johnson K Ratliff EA . Choosing and maintaining programs for sex education in schools: the CHAMPSS model. J Appl Res Child. (2011) 2:1–33.

[18] Peskin MF Hernandez BF Johnson K Addy RC Markham CM Shegog R . Sexual health education from the perspective of school staff: implications for adoption and implementation of effective programs in middle school. J Appl Res Child. (2011) 2:1–38.

[19] Peskin MF Hernandez B Gabay E Cuccaro P Li D Ratliff E . Using Intervention Mapping for program design and production of iCHAMPSS: an online-decision support system to increase adoption, implementation, and maintenance of evidence-based sexual health programs. Front Public Health. (2017) 5:203. 10.3389/fpubh.2017.00203 28848729 PMC5554483

[20] Bhargava HK Power DJ Sun D. Progress in web-based decision support technologies. Decis Support Syst. (2007) 43:1083–95. 10.1016/j.dss.2005.07.002

[21] Rogers EM. Diffusion of Innovations. 5th ed. New York, NY: Free Press (2003).

[22] Butterfoss D Kegler M Francisco V. Mobilizing organizations for health promotion: theories of organizational change. In: Glanz K, Rimer BK, Viswanath K, editors, Health Behavior and Health Education: Theory, Research and Practice, 4th edition. San Francisco, CA: Jossey-Bass (2008). p. 335–62.

[23] Bandura A. Social Foundations of Thought and Action. Englewood Cliffs, NJ: Prentice Hall (1986).

[24] Hernandez BF Peskin MF Shegog R Gabay EK Cuccaro PM Addy RC . iCHAMPSS: Usability and psychosocial impact for increasing implementation of sexual health education. Health Promo Pract. (2017) 18:366–80. 10.1177/1524839916682004 28420265

[25] Markham CM Torres J Craig Rushing S Gorman G Jessen C Gaston A . Usability and psychosocial impact of decision support to increase sexual health education in American Indian and Alaska Native communities. J Health Dispar Res Pract. (2018) 11:7.

[26] Walker SC Whitener R Trupin EW Migliarini N. American Indian perspectives on evidence-based practice implementation: results from a statewide tribal mental health gathering. Adm Policy Ment Health. (2015) 42:29–39. 10.1007/s10488-013-0530-4 24242820

[27] Fernandez ME ten Hoor G van Lieshout S Ruiter RAC Markham CM Biedas RS . Implementation Mapping: using intervention mapping to develop implementation strategies. Front Public Health. (2019) 7:158. 10.3389/fpubh.2019.00158 31275915 PMC6592155

[28] Bartholomew Eldredge LK Markham CM Ruiter RAC Fernández ME Kok G Parcel GS. Planning Health Promotion Programs: An Intervention Mapping Approach, 4th edition. San Francisco, CA: Jossey-Bass (2016).

[29] Wandersman A Duffy J Flaspohler P Noonan R Lubell K Stillman L . Bridging the gap between prevention research and practice: the interactive systems framework for dissemination and implementation. Am J Community Psychol. (2008) 41:171–81. 10.1007/s10464-008-9174-z 18302018

[30] Collins CB Edwards AE Jones PL Kay L Cox PJ Puddy RW . comparison of the interactive systems framework (ISF) for dissemination and implementation and the CDC division of HIV/AIDS prevention's research-to-practice model for behavioral interventions. Am J Community Psychol. (2012) 50:518–29. 10.1007/s10464-012-9525-7 22684737

[31] Wingood GM DiClemente RJ. The ADAPT-ITT model: a novel method of adapting evidence-based HIV interventions. J AIDS. (2008) 47(Suppl.1):S40–6. 10.1097/QAI.0b013e3181605df1 18301133

[32] Hawkins JD Catalano RF Arthur MW Egan E. Testing communities that care: the rationale, design and behavioral baseline equivalence of the community youth development study. Prev Sci. (2008) 9:178–90. 10.1007/s11121-008-0092-y 18516681 PMC2562862

[33] McKleroy VS Galbraith JS Cummings B Jones P Harshbarger C Collins C . Adapting evidence–based behavioral interventions for new settings and target populations. AIDS Educ Prev. (2006) 18(Supp.):59–73. 10.1521/aeap.2006.18.supp.59 16987089

[34] Wandersman A Imm P Chinman M Kaftarian S. Getting to outcomes: a results-based approach to accountability. Eval Program Plann. (2000) 23:389–95. 10.1016/S0149-7189(00)00028-8

[35] National Association of County and City Health Officials. Model Practice Database. Available online at: http://archived.naccho.org/topics/modelpractices/search.cfm (accessed March 1, 2022).

[36] Kang E Foster ER. Use of implementation mapping with community-based participatory research: development of implementation strategies of a new goal setting and goal management intervention system. Front Public Health. (2022) 10:834473. 10.3389/fpubh.2022.834473 35619816 PMC9127132

[37] Gray JS Rose WJ. Cultural adaptation for therapy with American Indians and Alaska Natives. J Multicult Couns Devel. (2012) 40:82–92. 10.1002/j.2161-1912.2012.00008.x25855820

[38] Chino M Debruyn L. Building true capacity: indigenous models for indigenous communities. Am J Public Health. (2006) 96:596–9. 10.2105/AJPH.2004.053801 16449598 PMC1470558

[39] Peters DH Adam T Alonge O Agyepong IA Tran N. Implementation research: what it is and how to do it. Br Med J. (2013) 347:bmj.f6753. 10.1136/bmj.f6753 24259324

[40] Shegog R Craig Rushing S Gorman G Jessen C Torres J . NATIVE-It's Your Game: adapting a technology-based sexual health curriculum for American Indian and Alaska Native youth. J Prim Prev. (2016) 38:27–48. 10.1007/s10935-016-0440-9 27520459

[41] Sacca L Craig Rushing S Markham CM Shegog S Peskin MF Hernandez B . Assessment of the reach, usability, and perceived impact of “Talking is Power”: a parental sexual health text-messaging service and web-based resource to empower sensitive conversations with American Indian and Alaska Native teens. Int J Environ Res Public Health. (2021) 18:9126. 10.3390/ijerph18179126 34501715 PMC8431363

[42] Roth IJ Tiedt MK Barnhill JL Karvelas KR Faurot KR Gaylord S . Feasibility of implementation mapping for integrative medical group visits. J Altern Complement Med. (2021) 27:S71–80. 10.1089/acm.2020.0393 33788606 PMC8035918

[43] Ibekwe LN Walker TJ Ebunlomo E Ricks KB Prasad S Savas LS . Using implementation mapping to develop implementation strategies for the delivery of a cancer prevention and control phone navigation program: a collaboration with 2-1-1. Health Promot Pract. (2022) 23:86–97. 10.1177/1524839920957979 33034213 PMC8032810

[44] National Institutes of Health. Dissemination and Implementation Research in Health. (2016). Available online at: https://grants.nih.gov/grants/guide/pa-files/PAR-16-238.html (accessed March 1, 2022).

[45] Kok G Gottlieb NH Commers M Smerecnik C. The ecological approach in health promotion programs: a decade later. Am J Health Promot. (2008) 22:437–42. 10.4278/ajhp.22.6.437 18677885

[46] Cook CR Lyon AR Locke J Waltz T Powell BJ. Adapting a compilation of implementation strategies to advance school-based implementation research and practice. Prev Sci. (2019) 20:914–35. 10.1007/s11121-019-01017-1 31152328 PMC8943907

[47] Lyon AR Cook CR Locke J Davis C Powell BJ Waltz TJ. Importance and feasibility of an adapted set of implementation strategies in schools. J Sch Psychol. (2019) 76:66–77. 10.1016/j.jsp.2019.07.014 31759470 PMC6876555

[48] Sacca L Shegog R Hernandez B Peskin M Craig Rushing S Jessen C . Barriers, frameworks, and mitigating strategies influencing the dissemination and implementation of health promotion interventions in Indigenous communities: a scoping review. Implement Sci. (2022) 17:18. 10.1186/s13012-022-01190-y 35189904 PMC8862215

[49] Fernandez ME Damschroder L Balasubramanian B. Understanding barriers and facilitators for implementation across settings. In: Weiner BJ, Lewis CC, Sherr K, editors, Practical Implementation Science. New York, NY: Springer Publishing Company (2023). p. 97–132. 10.1891/9780826186935.0005

[50] NPAIHB. Adolescent Health Tribal Action Plan. Available online at: https://www.healthynativeyouth.org/wp-content/uploads/2021/03/Adolescent-Health-Tribal-Action-Plan-2020-Final.org/wp-content/uploads/2021/03/Adolescent-Health-Tribal-Action-Plan-2020-Final. (accessed March 1, 2022).

[51] Alaska. Adolescent Health Tribal Action Plan. Available online at: https://www.healthynativeyouth.org/wp-content/uploads/2021/03/2020-AK-Adolescent-Health-Action-Plan-Final-10222020.pdf (accessed March 1, 2022).

[52] Fishbein M Ajzen I. Predicting and Changing Behaviour: The Reasoned Action Approach. New York, NY: Psychology Press (2010).

[53] Petty R Cacioppo JT. Communication and Persuasion: Central and Peripheral Routes to Attitude Change. New York, NY: Springer Science & Business Media (2012).

[54] Cummings TG Worley CG. Organization Development and Change. 10th ed. Mason, OH: South-Western Cengage Learning (2014).

[55] Eng E Rhodes SD Parker E. Natural helper models to enhance a community's health and competence. In: Di Clemente RJ, Crosby RA, Kegler MC, editors, Emerging Theories in Health Promotion Practice and Research. 2nd ed. San Francisco, CA: Jossey-Bass (2009). p. 303–30.

[56] Trevino N Gaston A. Healthy Native Youth: Virtual Adaptation Guide. Portland, OR: Northwest Portland Area Indian Health Board. Available online at: https://www.healthynativeyouth.org/wp-content/uploads/2021/02/8.-Virtual-Adaptation-Guide.pdf (accessed March 1, 2022).

[57] Holt-Lunstad J Uchino B. Social support and health behavior. In:Glanz K Rimer B Viswanath K, editors, Health Behavior: Theory, Research and Practice. 5th ed. San Francisco, CA: Jossey-Bass (2015). p. 183–204.

[58] Escobar-Chaves SL Shegog R Moscoso-Alvarez MR Markham C Tortolero-Luna G Peskin M . Cultural tailoring and feasibility assessment of a sexual health middle school curriculum: a pilot test in Puerto Rico. J Sch Health. (2011) 81:477–84. 10.1111/j.1746-1561.2011.00617.x 21740433

[59] Markham C Shegog R Leonard A Bui T Paul M. +CLICK: Harnessing web-based training to reduce secondary transmission among HIV-positive youth. AIDS Care. (2009) 21:622–31. 10.1080/09540120802385637 19444671 PMC2730352

[60] Powell BJ Beidas RS Lewis CC Aarons GA McMillen JC Proctor EK . Methods to improve the selection and tailoring of implementation strategies. J Behav Health Serv Res. (2017) 44:177–94. 10.1007/s11414-015-9475-6 26289563 PMC4761530

[61] Lewis CC Klasnja P Powell BJ Lyon AR Tuzzio L Jones S . From classification to causality: advancing understanding of mechanisms of change in implementation science. Front Public Health. (2018) 6:136. 10.3389/fpubh.2018.00136 29868544 PMC5949843

[62] Schlechter CR Del Fiol G Lam CY Fernandez ME Greene T Yack M . Application of community–engaged dissemination and implementation science to improve health equity. Prev Med Rep. (2021) 24:101620. 10.1016/j.pmedr.2021.101620 34976676 PMC8684008

[63] Pinto RM Park S Miles R Ong PN. Community engagement in dissemination and implementation models: a narrative review. Implement Res Pract. (2021) 1:1–18. 10.1177/2633489520985305 PMC9978697 37089998

[64] Kumpfer KL Alvarado R Smith P Bellamy N. Cultural sensitivity and adaptation in family-based prevention interventions. Prev Sci. (2002) 3:241–6. 10.1023/A:1019902902119 12387558

[65] Resnicow K Baranowski T Ahluwalia JS Braithwaite RL. Cultural sensitivity in public health: defined and demystified. Ethn Dis. (1999) 9:10–21. 10355471

[66] Kreuter M Lukwago SN Bucholtz DC Clark EM Sanders-Thompson VS. Achieving cultural appropriateness in health promotion programs: targeted and tailored approaches. Health Educ Behav. (2003) 30:133–46. 10.1177/1090198102251021 12693519

[67] Santisteban DA Muir-Malcom JA Mitrani JB Szapocznik J Integrating the study of ethnic culture and family psychology intervention science. In: Liddle HA, Santisteban DA, Levant RF, Bray JH, editors, Family Psychology: Science-Based Interventions. Washington, DC: American Psychological Association (2001). p. 331–51. 10.1037/10438-016

[68] Turner W. Cultural consideration is family-based primary prevention programs in drug abuse. J Prim Prev. (2000) 21:285–303. 10.1023/A:1007091405097

