# Supplementary figures and images for: The Healthy Native Youth Implementation Toolbox: Using Implementation Mapping to adapt an online decision support system to promote culturally-relevant sexual health education for American Indian and Alaska Native youth
Source: Front Public Health. 2022 Oct 31;10:889924. doi: 10.3389/fpubh.2022.889924 (PMC9659648; doi:10.3389/fpubh.2022.889924)

Supplemental Material. Healthy Native Youth Implementation Toolbox tools library by phase


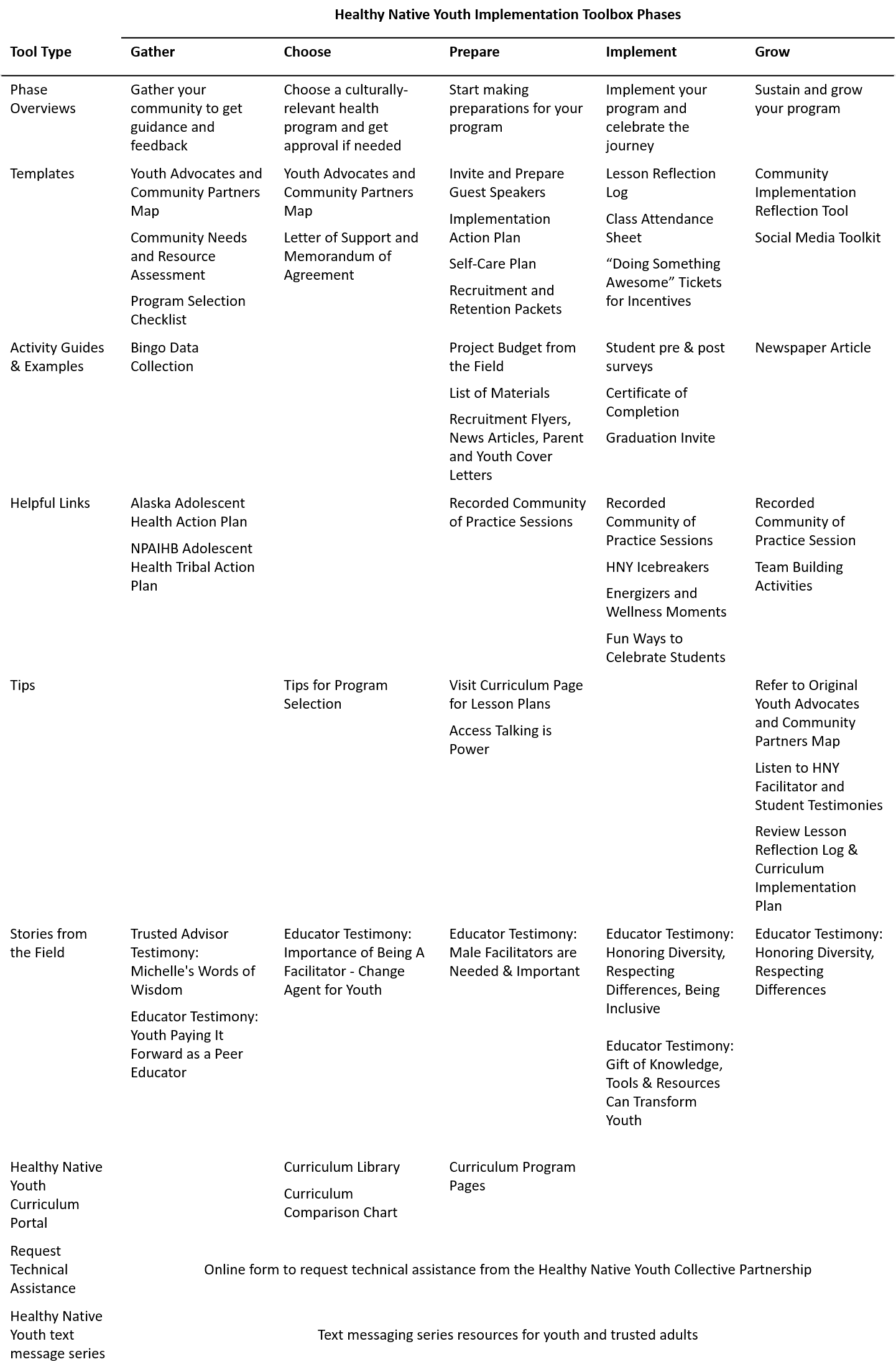

Supplement: Supplementary file 1 [file Table_1.DOCX]
