# Supplementary material for: The Healthy Native Youth Implementation Toolbox: Using Implementation Mapping to adapt an online decision support system to promote culturally-relevant sexual health education for American Indian and Alaska Native youth
Source: Front Public Health. 2022 Oct 31;10:889924. doi: 10.3389/fpubh.2022.889924 (PMC9659648; doi:10.3389/fpubh.2022.889924)
Supplement: Supplementary file 2 [file Data_Sheet_1.PDF]

Key Informant Interview Guide

Interviewer: Thank you for agreeing to participate in this interview. Our goal is to better understand how Native communities approach the process of adopting, implementing, and maintaining an adolescent sexual health education program. We want to hear about your experience - how you went about it, what helped, any challenges you encountered, and how you managed them. Any questions? Great, let's get started!

1. On a scale of 1-10, with 10 being the highest priority (or most important) and 0 being the lowest priority (or least important), how would you rate adolescent sexual health compared to other issues in your community? Why?

2. Which adolescent sexual health education program(s) have you used in your community?
   a. ___________________________________
   b. ___________________________________
   c. ___________________________________

3. Please describe your role within [name of organization] OR within the sexual health program. How did you personally get involved with the sexual health program (probe – had prior teaching experience? Was told to implement it? etc.)?

4. For the first program you mentioned (_______________________), please tell me about your experience getting that program up and running it in your community. Tell me from start to finish how you went about it. (*Let the interviewee tell their story uninterrupted…. this will allow them to share what was most memorable/salient).*

Thank you for sharing your story, you clearly have a lot of experience with this! I'd like to ask some follow-up questions, so that we can better understand each step in the process.

Adoption

5. First, please tell me how your community decided that it needed a sexual health education program, and how you chose a specific program. Take me through the steps…

   If not addressed here or previously, ask about:
   - Why the focus on sexual health?
     - Was there a particular incident that motivated the attention?
   - Who were the key stakeholders?
     - How were tribal elders involved?
     - Others? (Principals, parents, youth etc.)
   - How did you find the program (e.g., word of mouth, website, presentation)?
   - What was the deciding factor to select that program (e.g., student outcomes, cost, complexity of the program)?
   - Who, if anyone, had to give approval to use the program?
     - How did you go about getting that approval? How long did it take?

- Did you encounter any resistance? Who from?
    - How did you respond? What worked? What didn't work?

6. What advice would you give to others who are in the position of setting out to advocate for sexual health in their community? What are the top 3 things that could help them be successful?

<u>Implementation</u>

7. Next, please share how you went about running the program in your community. Take me through the steps…

If not addressed, ask about:

- Who was involved in running the program?
    - Community level (in what capacity?)
    - School/organization level (in what capacity?)
    - How did you identify the right people, including teachers?
- What level of involvement did Tribal Leaders while you were running the program? Did they know the program was taking place? Did they talk to you about the program while it was taking place?
- How prepared did you feel to deliver the program? Probe – Did you receive training on the program before implementation? Did you have adequate resources?
- On a scale of 1-10, with 0 being very easy and 10 being nearly impossible, how difficult was the sexual health program to deliver? Why? What were the challenges, how did you address them, what made things easier to deliver?

8. Based on your experience, what are the top 3 things to keep in mind when running a sexual health education program in Native communities?

<u>Maintenance</u>

9. Finally, please share how you went about keeping the program alive in your community.

If not addressed, ask about:

- What do you think has contributed to keeping this program alive in your community? Who were the key supporters?
- What resources were helpful in keeping the program alive (funding, administrative support, evaluation results, incentives, community improvement plan, community partners, etc.)?

<u>OR</u> If the program is no longer running:

- What led to the program not being delivered anymore? (*Probe for* lack of funding, lack of trained personnel, competing priorities, community resistance, another program taking its place, other factors?)

10. Based on your experience, what are the top 3 things to keep in mind when keeping a sexual health education program alive in Native communities?

11. *If the interviewee named more than one program:* You mentioned at the start that you've used other sexual health education programs besides (*first program*). Please tell me, how was the adoption and implementation process similar or different to (*first program*).

Native iCHAMPS

Our last questions ask about a new tool called Native iCHAMPS, which is a web-based decision support system to help Native communities adopt, implement and sustain evidence-based sexual health education program (*show them graphics of the website*).

12. How could this website be useful for Native communities?

13. What graphics or images would you suggest including?

14. Who should be the main audience for Native iCHAMPS?

15. What would motivate you to use Native iCHAMPS?

16. What type of technical support might you need to use Native iCHAMPS?

17. What would be the best ways to let people know about Native iCHAMPS?

That is all of our questions. Is there anything else we should know that we haven't asked about?

Thank you for sharing your story with us. Learning from your experience will help other Native communities address their youth's needs. Before we end, please complete this brief survey about your experience with sexual health education. We would also like to offer you a $20 e-gift certificate as a thank you for your participation. If you would like to receive the e-gift certificate, please give me the best email address to send it to:

____ (*write down email address)*

Thanks, that is everything! We really appreciate you taking the time to talk with us. We will provide you with a summary of the study when we are done.
